# Poly(*N*-vinylformamide)—Laponite XLG Nanocomposite Hydrogels: Synthesis and Characterization

**DOI:** 10.3390/gels12010031

**Published:** 2025-12-30

**Authors:** Paul Octavian Stănescu, Andrada Serafim, Anita-Laura Chiriac, Anamaria Zaharia, Raluca Şomoghi, Mircea Teodorescu

**Affiliations:** 1 Department of Bioresources and Polymer Science, Faculty of Chemical Engineering and Biotechnologies, National University of Science and Technology POLITEHNICA Bucharest, 1-7 Gh. Polizu Str, 011061 Bucharest, Romania; paul.stanescu@upb.ro (P.O.S.); andrada.serafim0810@upb.ro (A.S.); 2 Advanced Polymer Materials Group, National University of Science and Technology POLITEHNICA Bucharest, 1-7 Gh. Polizu Str, 011061 Bucharest, Romania; 3 National Institute for Research and Development in Chemistry and Petrochemistry—ICECHIM, Spl. Independentei 202, 060021 Bucharest, Romania; anita-laura.radu@icechim.ro (A.-L.C.); anamaria.zaharia@icechim.ro (A.Z.); r.somoghi@gmail.com (R.Ş.); 4 Faculty of Petroleum Technology and Petrochemistry, Petroleum and Gas University of Ploiesti, 39 Bucuresti Blvd., 100680 Ploiesti, Romania

**Keywords:** *N*-vinylformamide, Laponite XLG, nanocomposite hydrogel, NC gel, *N*,*N*’-methylenebisacrylamide

## Abstract

Novel highly compressible and stretchable nanocomposite (NC) hydrogels were obtained by the free radical polymerization of *N*-vinylformamide (NVF) in aqueous solution in the presence of Laponite XLG (XLG) as the crosslinker and 2,2′-azobis(2-methylpropionitrile) as the initiator. The expected composition of the NC hydrogels was proved by FTIR, TEM, XRD, and TGA analyses. Swelling degree (SD) and mechanical measurements showed that the properties of the PNVF NC hydrogels were largely different from those of both PNVF hydrogels covalently crosslinked by *N*,*N*’-methylenebisacrylamide (MBA) and equivalent poly(*N*-vinyl-2-pyrrolidone) (PNVP) NC hydrogels. After an initial fast swelling stage, the PNVF NC hydrogels displayed a slow, but steady, SD increase with time, unlike the MBA-crosslinked and NVP hydrogels, which exhibited a much smaller SD change during their second swelling stage. The mechanical testing of the synthesized hydrogels by uniaxial compressive and tensile measurements showed much higher compressibility (>90%) and stretchability (up to ≈840%) in the PNVF NC hydrogels than both PNVP and MBA-crosslinked PNVF hydrogels (compressibility < 80%; stretchability up to ≈114%). Cyclic compression tests revealed higher values for both elastic character and mechanical stability in the PNVF NC hydrogels in comparison to the MBA-crosslinked and PNVP ones. These different mechanical properties were explained by the PNVF NC gels possessing a network made of homogeneously distributed crosslinking sites and flexible polymer chains, thus avoiding extensive chain breakage up to larger stress values. The PNVF NC hydrogels described here may find applications for water purification, due to their high clay content, as well as in the biomedical field based on the biocompatibility of both the polymer and crosslinking agent.

## 1. Introduction

Poly(*N*-vinylformamide) (PNVF) is a water-soluble biocompatible polymer [1,2,3] that can be obtained by both the conventional [4,5] and controlled [6,7,8] free-radical polymerization of *N*-vinylformamide (NVF), a lower-toxicity structural isomer of acrylamide (AAm) [3,9,10,11]. In addition to its low toxicity, NVF displays both high water solubility and reactivity in (co)polymerization and subsequent polymer-analogous reactions as well as easier handling due to its room-temperature liquid state [1,9,11,12], which makes PNVF and its derivatives interesting replacements for PAAm and other water-soluble polymers in biomedicals, personal care products, water treatment, oil recovery, or papermaking fields [1,3,4,5,13].

An important application area of the water-soluble polymers is represented by hydrogels, which are described as chemically or physically crosslinked networks of hydrophilic polymer chains, displaying the ability to incorporate large quantities of water and aqueous solutions [14,15,16]. The field has continuously expanded since the first report on hydrogels by Wichterle and Lim in 1960 [17] due to hydrogels’ potential uses in many traditional and high-tech fields like medicine and pharmacy [18,19,20,21,22,23,24], cosmetics and hygiene products [25,26], catalysis [27], water purification and desalination [28,29,30,31], agriculture [32], microfluidics [29,33], actuators [29,34], biosensing and bioelectronics [29,35,36,37], oil and gas exploitation [38], membranes [39], and so on. Despite many useful properties, such as water content and elasticity, very valuable for applications in the biomedical field, early hydrogels displayed low mechanical properties, mainly due to their high water content combined with an inhomogeneous network [40,41], which hindered their application on a larger scale. Since then, researchers have developed many methods to obtain hydrogels with higher elastic modulus, stretchability, strength, and/or toughness, such as double-network hydrogels [42,43], slide-ring (topological) hydrogels [44], tetra-PEG hydrogels [40,45], nanocomposite (NC) hydrogels (gels) [46], etc., which have led to a spectacular development of the hydrogel application field.

One of the first discovered categories of hydrogels with improved mechanical properties is represented by NC hydrogels, which are able to resist high deformations of various types [46]. NC hydrogels, initially reported by Haraguchi and Takehisa [47] in 2002, have been most often obtained through the free-radical (co)polymerization of a vinyl monomer of the acrylamide type, such as *N*-isopropylacrylamide [47,48], acrylamide [49,50,51], *N*,*N*-dimethylacrylamide [52,53], *N*,*N*-diethylacrylamide [54], *N*-acryloylglycinamide [55], or 4-acryloylmorpholine [56], by using Laponite clay as the only crosslinker. Very few non-acrylamide monomers have been reported as forming NC gels so far: 2-dimethylaminoethyl methacrylate [57], 2-(2-methoxyethoxy)ethyl methacrylate-oligo(ethylene glycol) methacrylate comonomers [58], and *N*-vinyl-2-pyrrolidone (NVP) [59,60].

Laponite is a synthetic hectorite-like clay belonging to the smectite group, with the Na^+^_0.7_[(Si_8_Mg_5.5_Li_0.3_)O_20_(OH)_4_]^−0.7^ empirical formula, made up of hydrophilic layered crystal stacks that convert to isolated discs (platelets) of 25–30 nm diameter and about 1 nm thickness after dispersion in water [61,62,63]. The discs contain negative charges on the surface due to the partial substitution of Mg^2+^ ions with Li^+^ ions and positive charges on the edges because of the protonation of the exposed OH groups contained. The electrostatic interaction of the original Laponite (Laponite XLG/RD) platelets in water leads to a “house-of-cards” type of structure, which may result in the gelation of the aqueous dispersion if the clay concentration is above 2% [61,62]. To avoid the gel formation at higher concentrations of clay dispersion, the positive charges are neutralized with pyrophosphate anions (Laponite XLS/RS) [64]. Laponite was reported as being non-cytotoxic below a certain concentration and degradable in water at pH less than 10, i.e., the isoelectric point of Laponite, in about 20–50 days [61,62,63]. Both types of Laponite clay were used for the preparation of NC gels [60].

The NC hydrogel network, described for the acrylamide-type monomers, consists of polymer chains connected by individual Laponite discs acting as multifunctional crosslinkers. Each clay disc is surrounded by a layer, about 1 nm thick, of polymer chains that interact with the disc surface through hydrogen bonds formed between the clay Si-O groups and the amide functionalities belonging to the polymer. The chains connecting the Laponite platelets result from a termination-by-combination reaction between macroradicals growing from the surface of the discs, where molecules of the monomer and initiator are initially adsorbed. In the final, a hydrogel structure with flexible polymer chains and homogeneously distributed multifunctional crosslinking points results, which is able to avoid stress concentration on a small number of chains during deformation, thus greatly improving the mechanical properties [46,65].

Both hydrogels and micro- and nanogels of NVF homo- and copolymers have already been reported in the literature [3,9,11,13,66,67,68,69,70], their crosslinking being carried out thermally, in the absence of any chemical crosslinker [69] or through the radical copolymerization of NVF/comonomers with bisvinyl crosslinkers with either acrylic [11,70] or *N*-vinylamide [3,9,66,67,68] polymerizable groups. Due to the close reactivities of the polymerizable groups involved in the process, the bisvinylamide crosslinkers have led to PNVF hydrogels with a more homogeneous network, especially from the chain length point of view, and, therefore, to better mechanical properties than those of the corresponding hydrogels obtained with acrylic crosslinkers [9].

Besides the PNVF hydrogels obtained through copolymerizing NVF with crosslinking monomers of similar reactivity, one would expect that NVF, which is a *N*-vinylamide monomer belonging to the same class as NVP, would be able to produce NC hydrogels with good mechanical properties as well. To the best of our knowledge, the present paper describes, for the first time, the synthesis and properties of the NC hydrogels obtained through the free-radical polymerization of NVF in aqueous solution in the presence of Laponite XLG as the crosslinking agent. We will show within this paper that Laponite XLG is a very good crosslinking agent for the synthesis of PNVF hydrogels with a more homogeneous network and excellent both compressibility and stretchability, in comparison with *N*,*N*’-methylenebisacrylamide-crosslinked NVF hydrogels. The PNVF nanocomposite hydrogels reported within this paper have also displayed much better compressibility and stretchability as compared to equivalent poly(*N*-vinyl-2-pyrrolidone) (PNVP) NC hydrogels.

One should mention that NC hydrogels containing NVF monomer units have been obtained previously by copolymerizing an acrylamide–acrylonitrile–NVF monomer mixture (6:2:2 mole ratio) in the presence of both *N*,*N*’-methylenebisacrylamide and Laponite XLG as the crosslinkers [13]. However, while acrylamide was already known to be producing NC gels by interaction with Laponite [49,50,51], the contribution of the NVF unit–Laponite interactions to the crosslinking process was not clearly stated or proven within that paper.

Based on the literature data, we may assume that the PNVF NC hydrogels described here may find applications for water purification due to their high clay content, similarly with some PNVP-XLG NC hydrogels [71], as well as in the biomedical field by taking into account the biocompatibility of the main hydrogel components, i.e., PNVF [1,2,3] and Laponite [61,62,63].

## 2. Results and Discussion

### 2.1. Synthesis and Structure of Hydrogels

**Figure 1:**
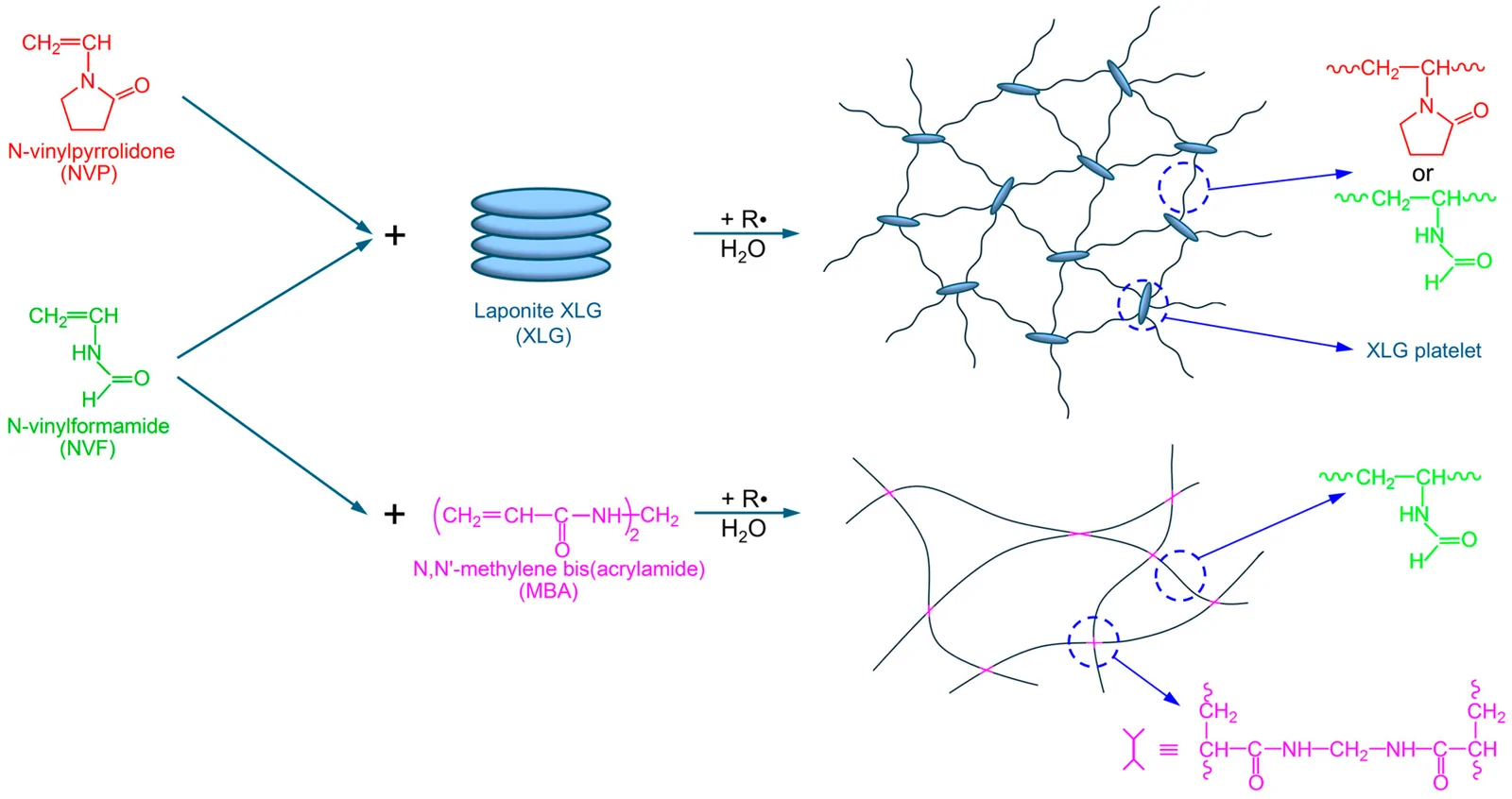
The chemical structures of monomers, crosslinking agents, and synthesized hydrogels.

The PNVF nanocomposite hydrogels were prepared in aqueous solution by the free-radical polymerization of NVF in the presence of Laponite XLG as the crosslinker and AIBN as the initiator. We did not use a persulfate to initiate the polymerization, despite its water solubility, because of reports indicating persulfates as “unreliable” in the case of NVF radical polymerization [4]. AIBN, although water-insoluble, has been successfully employed previously for the preparation of other NC hydrogels as well [53,60,71]. PNVF hydrogels were covalently crosslinked by means of MBA, with or without XLG added, and PNVP nanocomposite hydrogels were prepared under similar experimental conditions for comparison. The chemical structures of monomers, crosslinking agents, and synthesized hydrogels are displayed in Figure 1.

Attempts to use (triethylene glycol) divinyl ether as a crosslinker led to soluble PNVF polymer only although, in the case of NVP, the same crosslinking agent produced hydrogels with very good properties previously [59]. To obtain hydrogels with various properties, NVF concentrations of 10 wt%, 15 wt%, and 20 wt% of the whole polymerization mixture, XLG/NVF weight ratios of 5, 15, 20, and 25%, and MBA/NVF mole ratios of 1, 2, and 4% were employed. The AIBN concentration was set to 0.25 mol% based on NVF. The NVF concentration was limited to 20 wt% in order to avoid the formation of covalently crosslinked polymer as a consequence of various free-radical processes occurring during the polymerization [72]. Also, the XLG concentration was limited in order to avoid an excessive increase in viscosity of the clay aqueous suspension.

**Figure 2:**
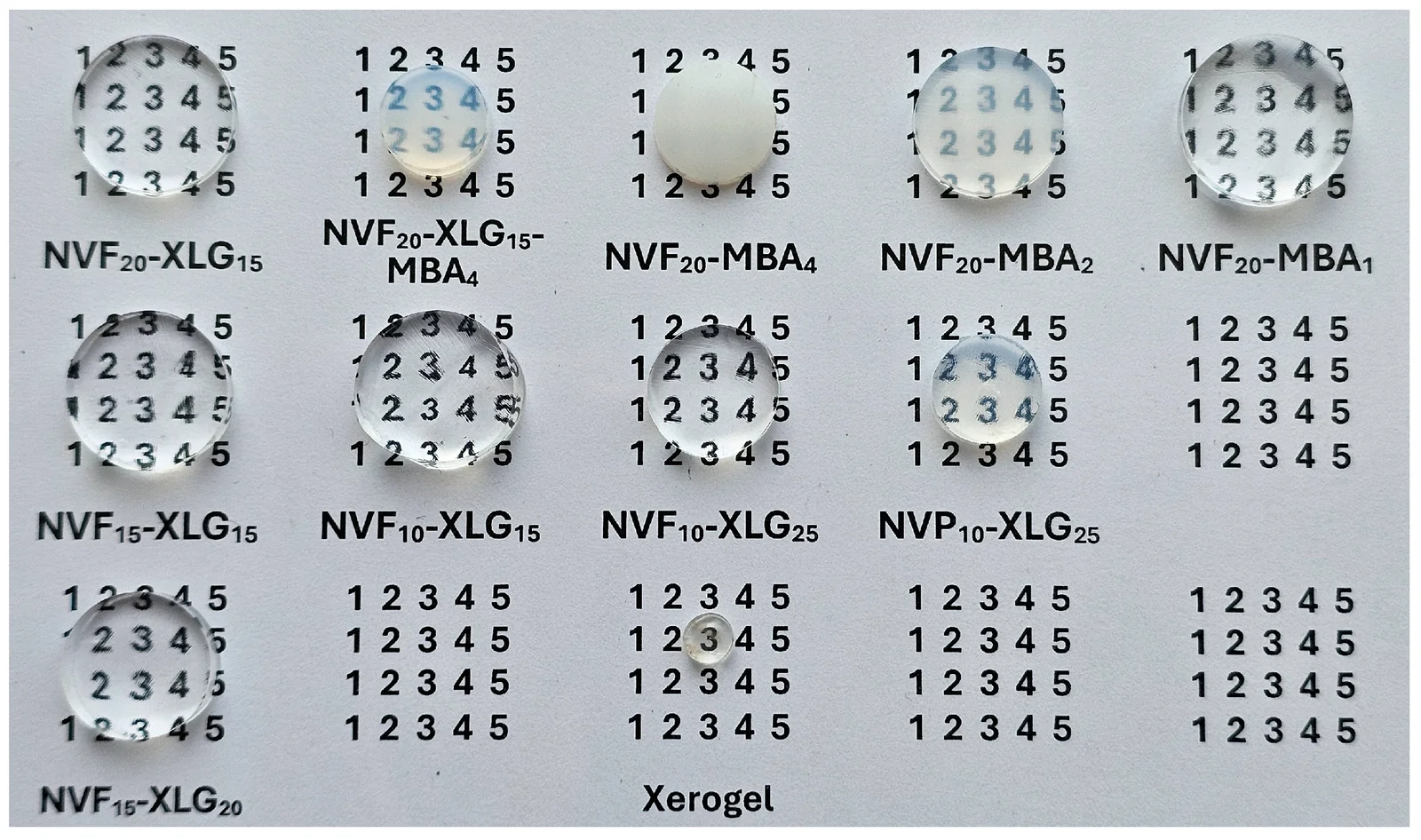
The aspect of the synthesized hydrogels after swelling in DW, at room temperature, for 7 days. These hydrogels were obtained from previously polished xerogels with a thickness of 1 ± 0.05 mm.

The pre-hydrogel liquid mixtures were heated at 50 °C for 24 h, resulting in monomer conversions over 95% in all cases, while the gel fractions (GF) ranged between 71.5% and 94%, depending on both the monomer and crosslinking agent type and amount (as presented in the Section 4). The gel fractions recorded in the case of XLG-containing hydrogels were generally larger than those for the MBA-crosslinked hydrogels (except for NVF_15_-XLG_5_ because of the low amount of crosslinking agent), pointing at a more homogeneous network, with a lower amount of defects, in the first case [73]. The lower GF in the case of MBA-only-crosslinked hydrogels may be rationalized through the much higher reactivity of MBA in the copolymerization reaction with NVF, as suggested by the copolymerization reactivity ratios of the acrylamide (AM)-NVF monomer pair (r_AM_/r_NVF_ = 0.51/0.046 [74]). This determined a much faster consumption of MBA in comparison with NVF, leading to the formation of soluble PNVF polymer during the final polymerization stages. This explanation was supported by the opaque/cloudy aspect of the swollen MBA-containing hydrogels (Figure 2), except for NVF_20_-MBA_1_, which was indicative of a multiphase material. The multiphase structure of these hydrogels was made up of higher-density regions with a larger MBA concentration, which resulted during the first stages of the polymerization process, surrounded by regions with a lower crosslinking density/MBA content, formed during the more advanced polymerization stages [50]. The NVF_20_-MBA_1_ hydrogel was clear (Figure 2), unlike all the other MBA-containing hydrogels, because of the lower MBA/NVF mole ratio in the pre-gel solution, which did not allow a too-fast MBA consumption in comparison with NVF. Therefore, higher-density regions did not form in this case, and phase separation within the swollen hydrogel did not occur any longer.

All swollen NVF-XLG hydrogels were clear, without any sign of phase separation, irrespective of the NVF or XLG concentrations within the pre-gel solution, while the appearance of NVF_20_-XLG_15_-MBA_4_ was cloudy, that is, intermediate between those of the fully transparent NVF_20_-XLG_15_ and fully opaque NVF_20_-MBA_4_. The NVP_10_-XLG_25_ hydrogel was slightly cloudy, unlike the NVF hydrogel with the same composition, probably because of the presence of a larger number of non-exfoliated clay particles [60].

**Figure 3:**
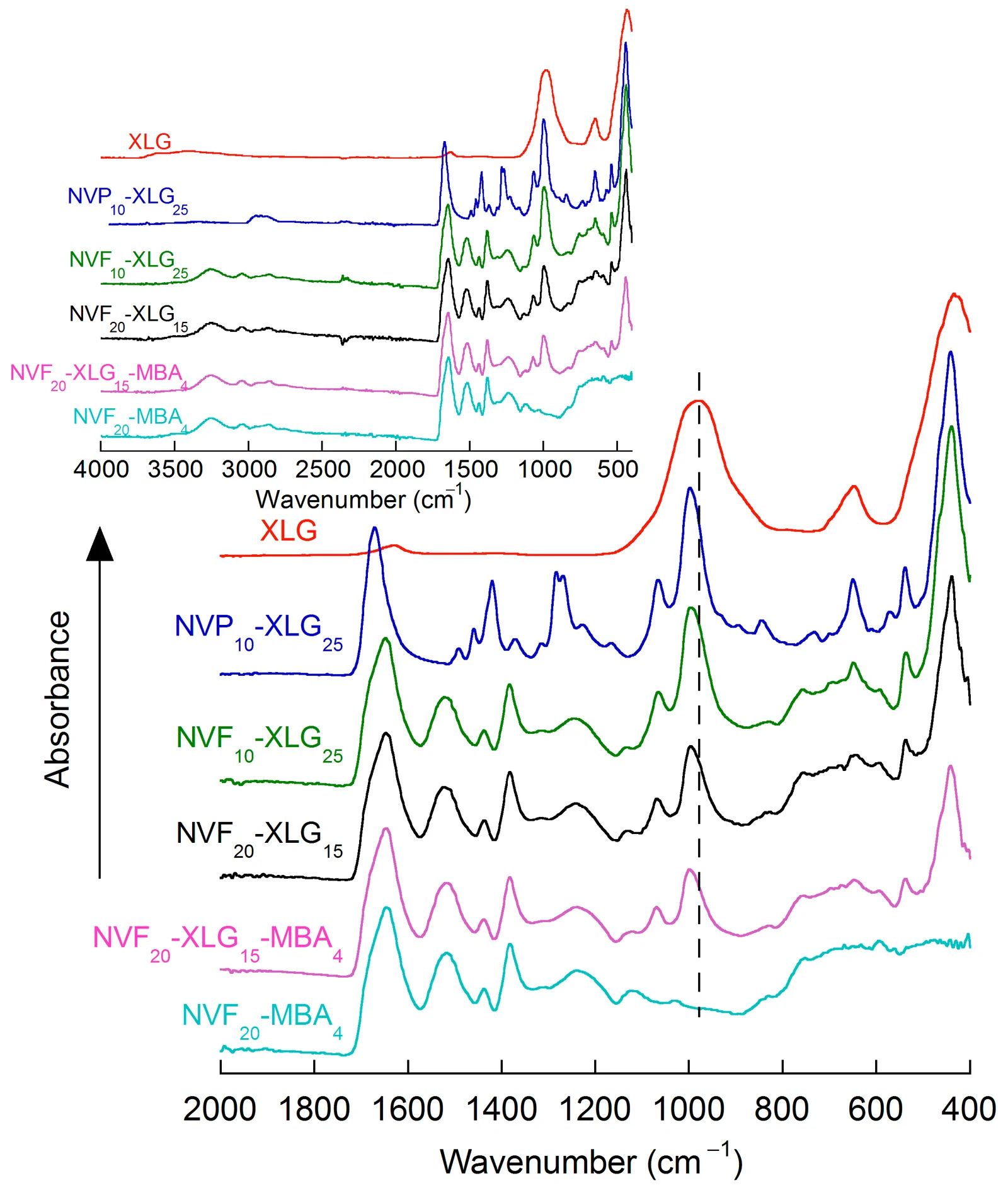
FTIR spectra of the hydrogels synthesized. To improve the figure aspect, the spectra were normalized against the band at 1647 cm^−1^.

The FTIR spectra of the xerogels (Figure 3 and Figure S1—Supporting Information) revealed the presence of the XLG band (Si-O stretching vibration, originally at 978 cm^−1^ [75]) in the NC xerogels, split into two bands shifted to larger wavenumbers due to the clay interactions with both PNVF and PNVP chains, confirming previous findings [60]. Also, the FTIR spectra of all PNVF nanocomposite xerogels, as well as that of NVF_20_-MBA_4_, contained the important bands of the PNVF matrix: amide I (>C=O stretching) at about 1647 cm^−1^, amide II (N-H bending and C-N stretching) at ≈1520 cm^−1^, N-H stretching at ≈3260 cm^−1^, CH_2_ stretching at about 1439 cm^−1^, and the C-N stretching of the primary amide (O=CH-NH-) at ≈1240 cm^−1^ [10,76,77,78]. The characteristic bands of the PNVP matrix—amide I at 1672 cm^−1^, C-H_n_ deformations at 1419–1490 cm^−1^, and N-C stretching vibration at 1283 cm^−1^ [60]—could be seen, beside the XLG bands, in the FTIR spectrum of the NVP_10_-XLG_25_ NC gel.

**Figure 4:**
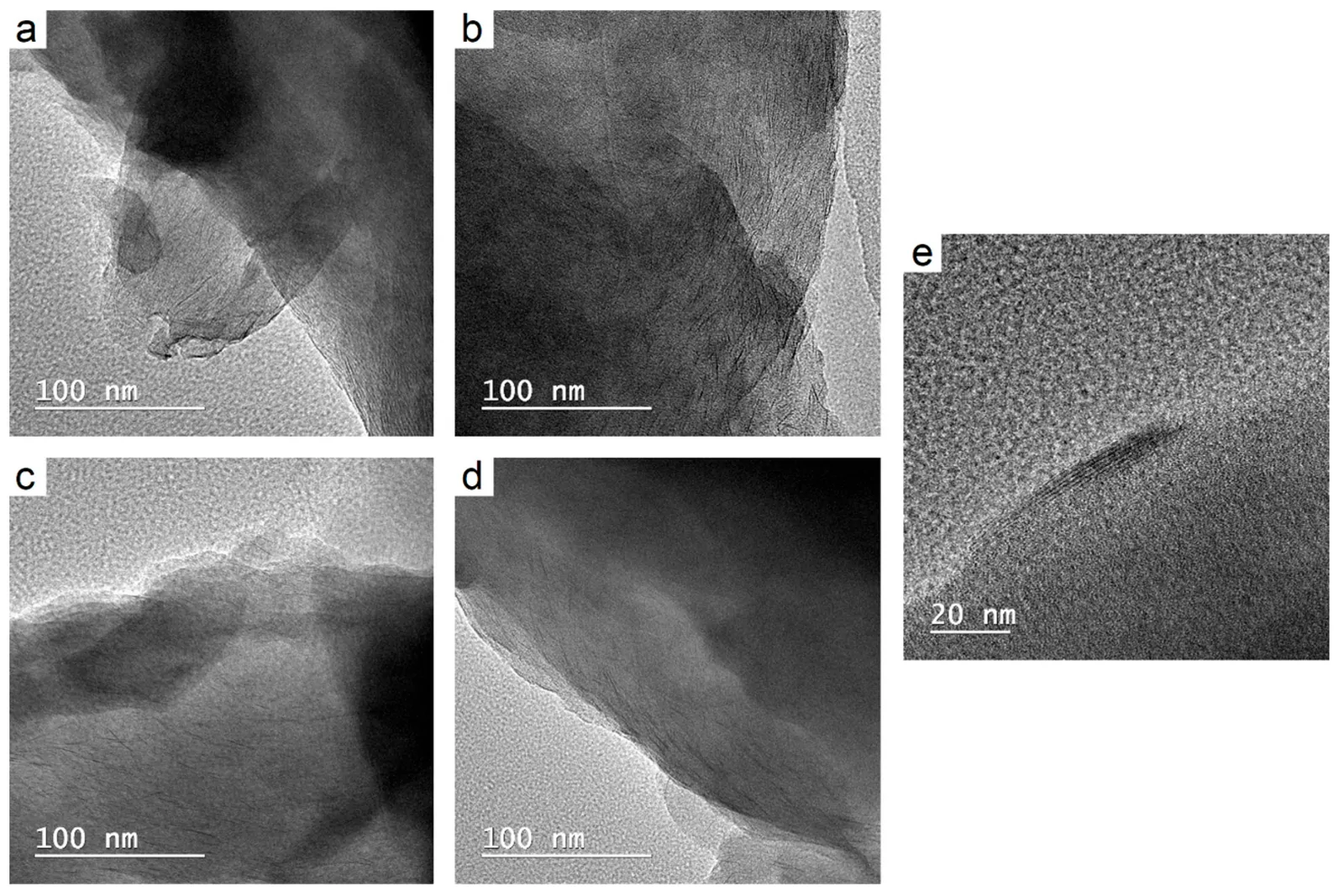
TEM images for some of the synthesized nanocomposite hydrogels. (**a**) NVF_10_-XLG_25_; (**b**) NVP_10_-XLG_25_; (**c**) NVF_20_-XLG_15_; (**d**) NVF_20_-XLG_15_-MBA_4_; (**e**) non-exfoliated Laponite stack in NVF_20_-XLG_15_-MBA_4_.

The structure of the synthesized NC hydrogels was additionally investigated by TEM, XRD, and TGA analyses, all of them demonstrating the presence of the clay within the crosslinked material. Thus, the TEM images proved the exfoliation of the most part of the clay within the hydrogel (Figure 4a–d), isolated platelets with a random orientation being visible inside the nanocomposite hydrogels. However, the analysis indicated that a small part of the clay was still present as non-exfoliated stacks (Figure 4e).

**Figure 5:**
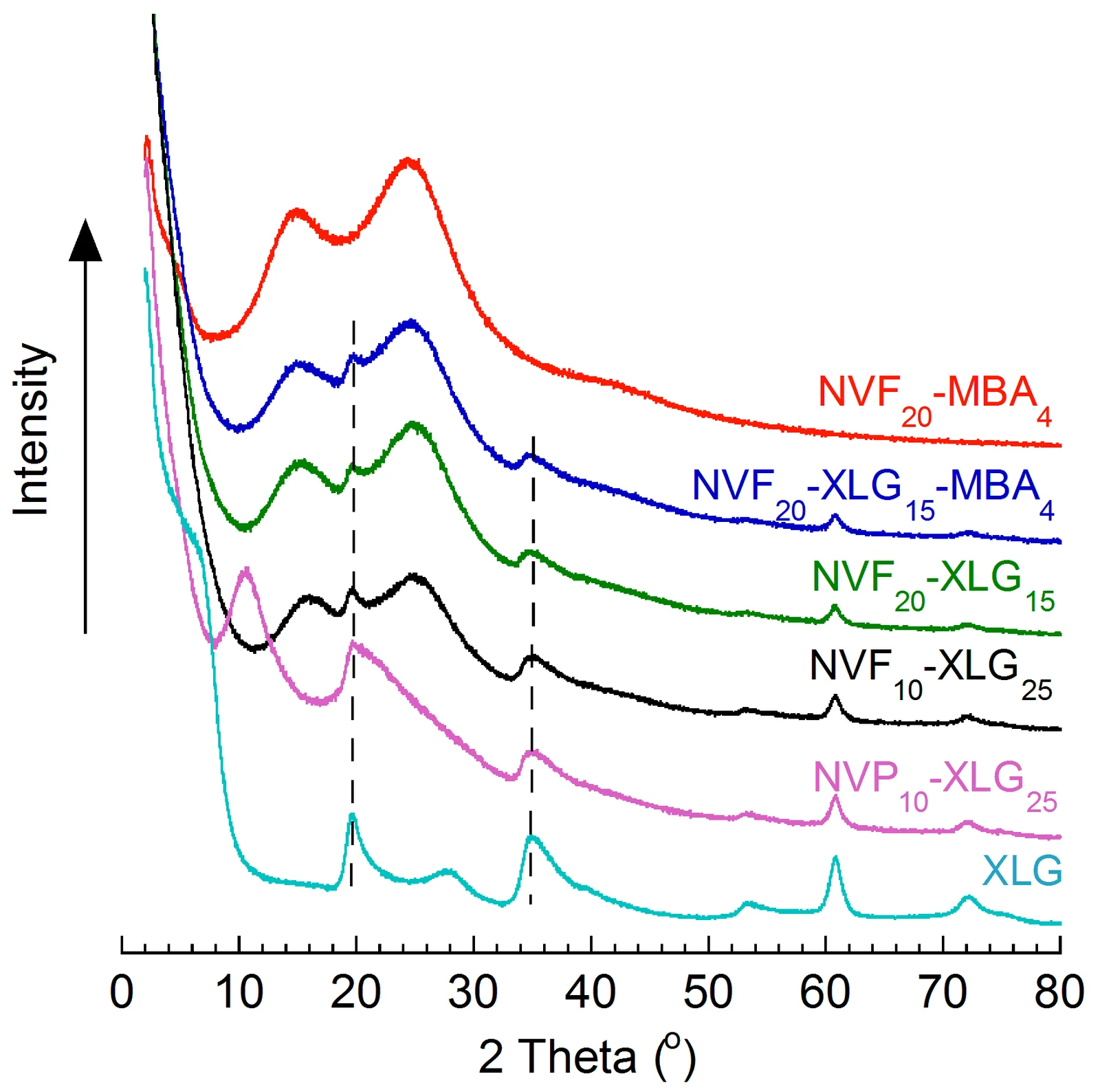
Comparison of the XRD patterns of Laponite XLG, some of the NC hydrogels synthesized, and the PNVF matrix.

The conclusions of the TEM investigation were supported by the XRD measurements (Figure 5 and Figure S2). The X-ray diffractograms of the synthesized NC hydrogels contained some small peaks belonging to non-exfoliated XLG stacks, in agreement with the results of the TEM analysis. However, the small intensity of these XLG peaks confirmed that most of the clay was exfoliated within the material. In addition to this, all NC hydrogel diffractograms displayed two broad peaks, characteristic of the patterns of the corresponding polymer matrix, i.e., PNVP (2θ ≈ 11° and 2θ ≈ 21° [60]) and PNVF (2θ ≈ 14.5° and 2θ ≈ 24° [79]).

**Figure 6:**
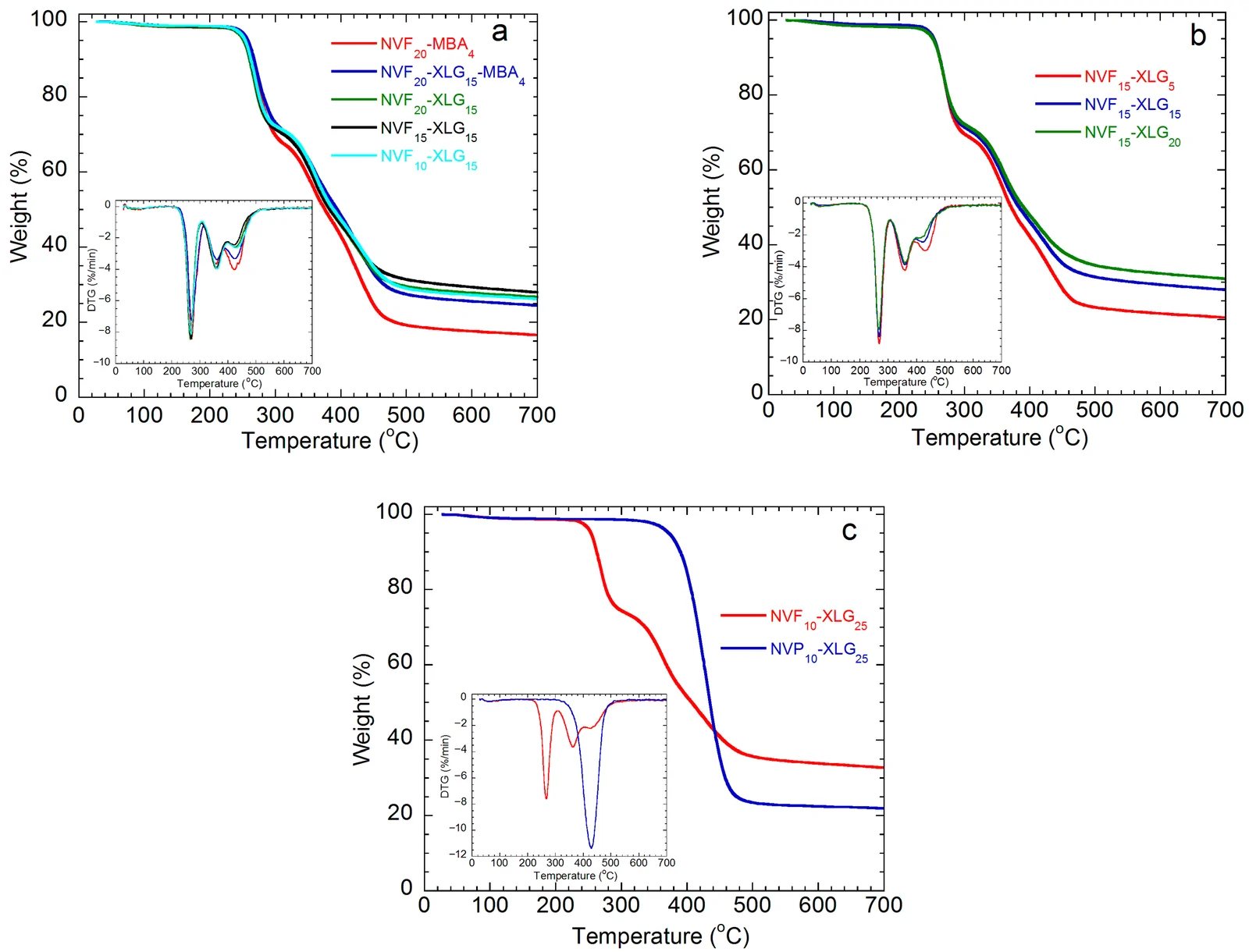
TGA and DTG (inset) plots of the hydrogels synthesized: (**a**) influence of monomer concentration; (**b**) influence of XLG concentration; (**c**) comparison between NVF and NVP nanocomposite hydrogels.

The TG analysis of the xerogels obtained showed a three-step decomposition in the case of the NVF hydrogels (Figure 6), in agreement with the literature data [80]. The first mass loss stage, occurring within the 200 °C–≈310 °C interval, was ascribed to the NHCHO group elimination from the polymer chains. The second decomposition step (≈310 °C–≈395 °C) was related to carbon chain cracking events resulting in the formation of aromatic structures while the third one (≈395 °C–≈520 °C) was due to condensed aromatic structure formation [80]. One should notice that an electrical conductive carbon [80] residue of about 16% resulted at 700 °C in the case of the XLG-free NVF_20_-MBA_4_ hydrogel (Figure 6a). The presence of the clay within the hydrogel led to an additional increase in the residue amount (Figure 6a), which was higher for larger XLG/NVF mass ratios (Figure 6b). For equal XLG/NVF weight ratios (15 wt%), very similar solid residue percentages, i.e., 25–28%, were obtained (Figure 6a). One should mention that the nanocomposite thermal stability was not affected by changing the clay amount (Figure 6a,b).

Unlike the NVF hydrogels, a single-stage decomposition occurred in the case of NVP_10_-XLG_25_ (Figure 6c), while the resulting residue amount was practically equal to the nanocomposite clay content, in agreement with previously published data [60].

### 2.2. Swelling Degree Investigations

**Figure 7:**
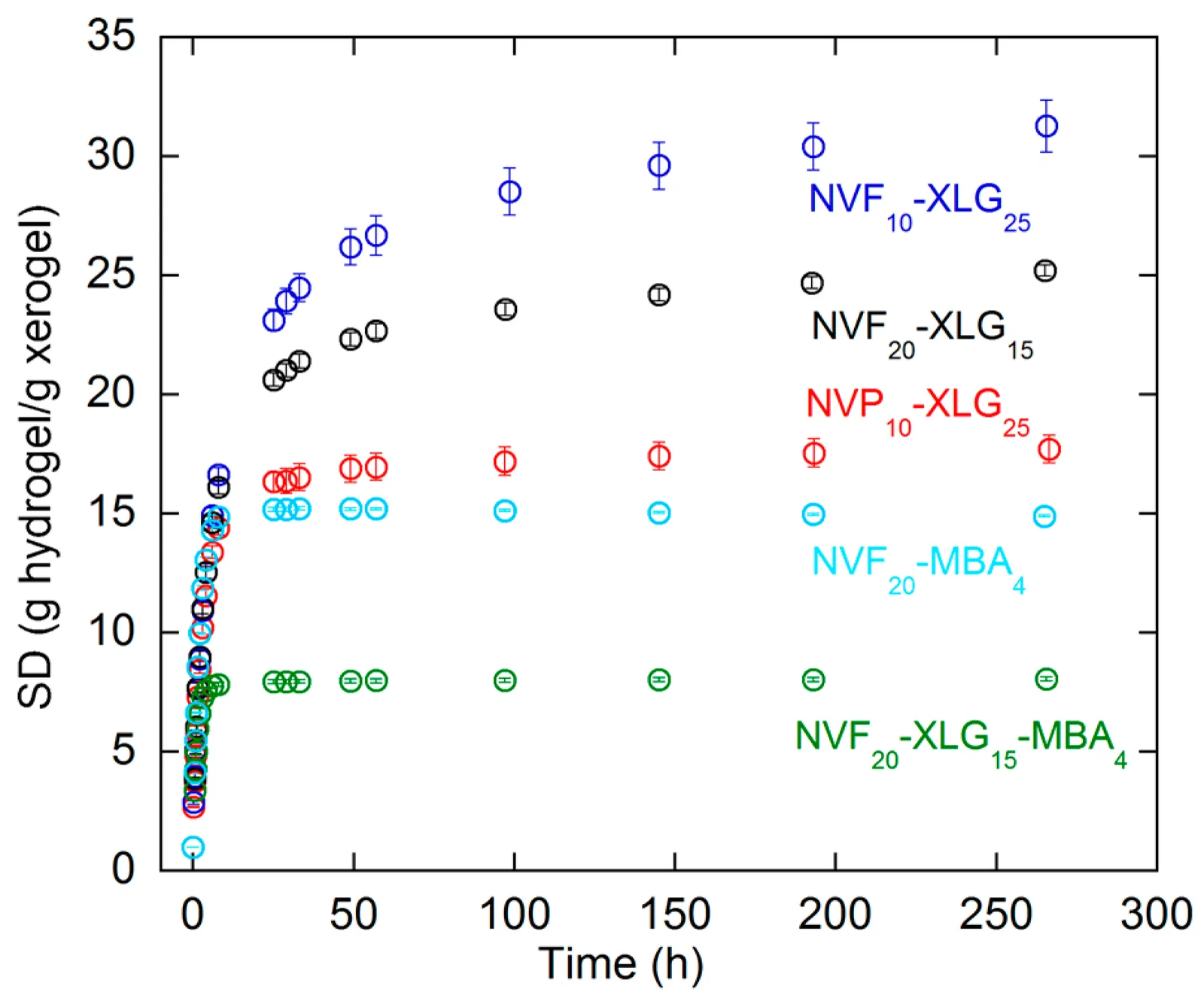
Swelling degree vs. time plots showing the dependence of the swelling curve shape on the crosslinking agent.

Swelling degree (SD) vs. time experiments for the hydrogels synthesized were carried out in DW at 25 ± 1 °C (Figure 7 and Figure S3a–e). The results obtained showed a different swelling behavior in the XLG-crosslinked hydrogels in comparison with the MBA-crosslinked ones. After an initial fast swelling stage, common for both hydrogel types, a second step characterized by a slow but steady increase in SD with time occurred in the case of the nanocomposite hydrogels while a very slight SD decrease with time was noticed for the covalently crosslinked hydrogels on the time interval investigated, i.e., about 300 h (Figure 7). In the case of NVF_20_-XLG_15_-MBA_4_ hydrogel, crosslinked by means of both XLG and MBA, the second swelling step consisted of a slow SD increase with time as well, but much slower than in the case of the corresponding hydrogel without MBA, i.e., NVF_20_-XLG_15_. Also, the SD increase rate was lower for the PNVP hydrogel in comparison with its PNVF counterpart (see NVP_10_-XLG_25_ vs. NVF_10_-XLG_25_, Figure 7). No equilibrium swelling stage was reached for any of the hydrogels tested during the testing time interval.

We do not have a clear explanation for the continuous increase with time in the SD of the XLG-containing hydrogels during the second swelling stage. A possible reason may have been the sluggish decrease in the hydrogel crosslinking density, determined by the gradual XLG degradation/dissolution in DW [61,62,63], and/or the slow basic hydrolysis of the vinylformamide units [81] triggered by the basic pH inside the hydrogel produced by the clay reaction in DW (pH = 9.6 at 1.5 wt% XLG in aqueous dispersion [82]). In the second case, the crosslinking density would be reduced by the replacement of the formamide groups involved in the XLG–PNVF chain bond formation by NH_2_ or OH groups [81], unable to form physical bonds with the clay. The FTIR analysis of the xerogel powders obtained after keeping the hydrogels in DW at room temperature for more than one year revealed a slight intensity decrease in the XLG bands and no additional bands in the FTIR spectra (Figure S4), which seems to point at the first supposition, i.e., a decrease in the crosslinking density as a consequence of the clay degradation. However, additional TGA investigations targeting the clay content of the hydrogels failed to indisputably support this conclusion.

The swelling experiments also showed that SD depended on the crosslinking degree of the hydrogel and monomer concentration in the pre-hydrogel solution [83], as well as on the polymer’s hydrophilic character [84], as expected. Thus, SD was lower for hydrogels with higher crosslinking degrees, i.e., higher crosslinking agent concentration in the pre-hydrogel solution (SD_NVF20-XLG15-MBA4_ < SD_NVF20-XLG15_, SD_NVF20-XLG15-MBA4_ < SD_NVF20-MBA4_, Figure 7; SD_NVF20-MBA4_ < SD_NVF20-MBA2_ < SD_NVF20-MBA1_, Figure S3a; SD_NVF15-XLG20_ < SD_NVF15-XLG15_, Figure S3b; SD_NVF10_-XLG_25_ < SD_NVF10-XLG15_, Figure S3c) or larger monomer concentration in the pre-hydrogel solution (SD_NVF20-XLG15_ < SD_NVF15-XLG15_ < SD_NVF10-XLG15_, Figure S3d), as well as in the case of the hydrogel made up of the less hydrophilic polymer (SD_NVP10-XLG25_ < SD_NVF10-XLG25_, Figure S3e), due to the lower hydrophilic character of the NVP structural unit in comparison with the NVF one, as demonstrated by the chemical structures (Figure S3e). These findings were in agreement with the sizes (diameters) of the hydrogels swollen at room temperature for 7 days, as seen in Figure 2. However, an apparent disagreement may be noticed in the case of NVF_10_-XLG_15_, NVF_15_-XLG_15_, and NVF_20_-XLG_15_, whose diameters appeared as being practically equal (Figure 2), despite the obvious SD differences (Figure S3d). This can be explained by the different heights of the swollen hydrogels (Figure S5), whose order followed that of the SDs.

### 2.3. Mechanical Properties of Hydrogels

**Figure 8:**
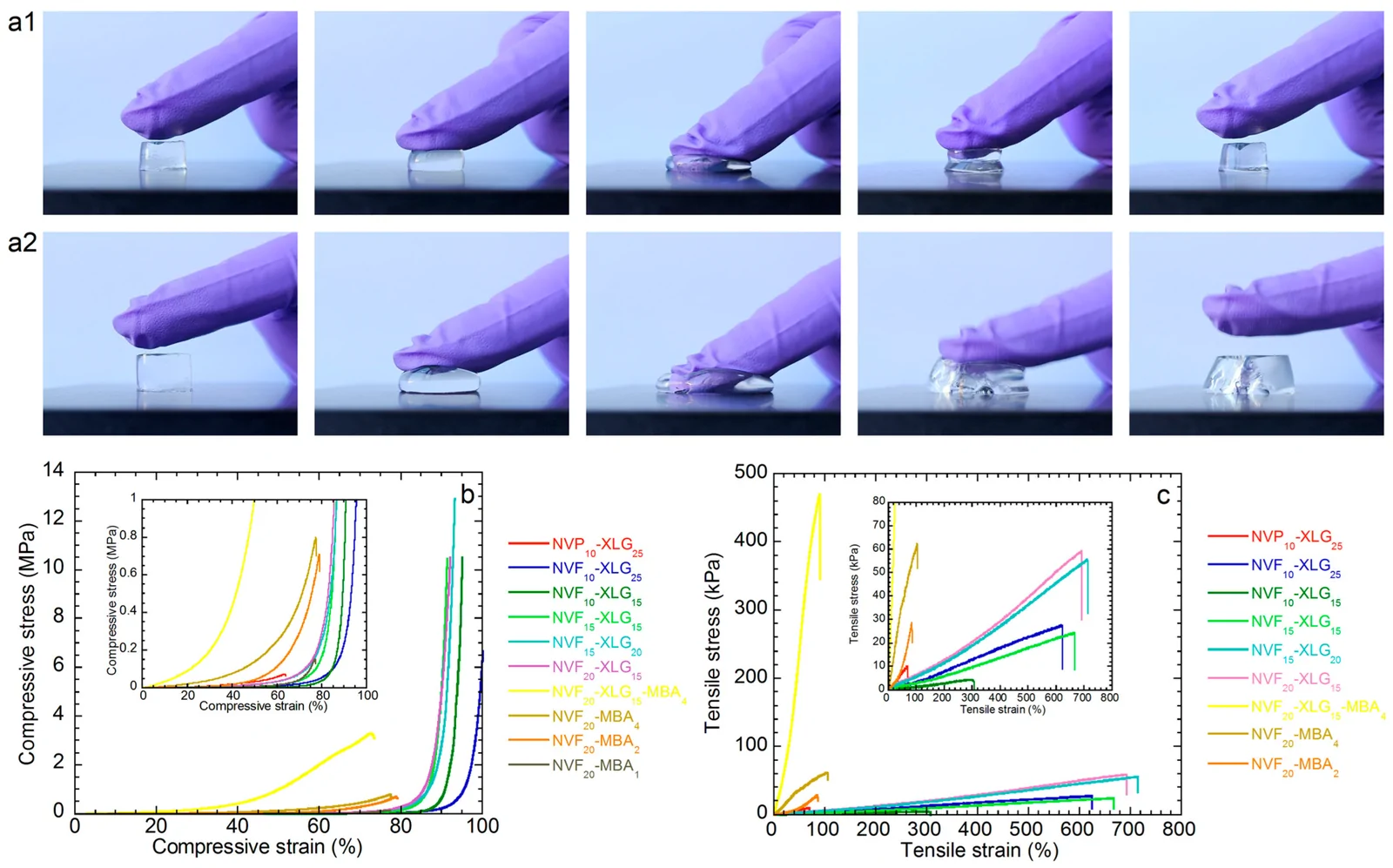
Mechanical properties of the hydrogels synthesized. (**a**) The different behavior of the NVF-XLG hydrogels vs. the MBA-containing ones under compression by hand. (**a1**) NVF_10_-XLG_25_; (**a2**) NVF_20_-MBA_1_. (**b**) Typical compressive stress–strain curves. The curves with the closest parameters to the average values shown in Table 1 are displayed. (**c**) Typical tensile stress–strain curves. The curves with the closest parameters to the average values shown in Table 2 are displayed.

The NVF-XLG nanocomposite hydrogels proved to be very soft and highly compressible when pressed by hand, resisting without damage to full squeezing (Figure 8a1), unlike the MBA-containing hydrogels that were easily crushed by hand if soft enough (Figure 8a2). This behavior difference between the two hydrogel types was better revealed by measuring their mechanical properties by means of unconfined uniaxial compressive tests, tensile tests, and cyclic compression tests.

The compression tests were performed at room temperature on hydrogel cylinders, swollen in DW immediately after synthesis for 11–13 days, whose heights were comparable to their diameters. Both the elastic (E_comp_) and shear (G) moduli and their ratio (E/G), the ultimate compressive stress (compression strength, τ_c,max_) and the associated ultimate compressive strain (1 − λ)_max_, and the compressive toughness (T_c_) were determined as mean values of four-measurement sets (Table 1). The results showed that unlike the NVF hydrogels containing covalent crosslinking sites, i.e., NVF_20_-XLG_15_-MBA_4_ and NVF_20_-MBA_1–4_, the NVF NC hydrogels crosslinked by XLG only did not display a fracture point up to at least 95% strain (sample NVF_10_-XLG_25_, Figure 8b and Figure S6a–c), but they irreversibly deformed at strain values larger than 90% (Figure S7). That is why we considered 90% strain as the value corresponding to the compression strength and toughness in the case of these hydrogels (Table 1), even though the compression measurements were generally stopped at strain values larger than 90%. The NVP_10_-XLG_25_ nanocomposite hydrogel, although crosslinked by XLG only, behaved similarly with the MBA-crosslinked hydrogels (Figure 8b and Figure S6c), in agreement with previous reports [60]. For the MBA-crosslinked hydrogels and NVP_10_-XLG_25_, the ultimate compressive stress (Table 1) was equal to the stress value corresponding to hydrogel failure.

The mechanical properties of the synthesized hydrogels obtained by compression tests, together with the corresponding SDs, are displayed in Table 1. Both E_comp_ and G were determined, and the results showed that they strongly depended on the hydrogel SD, i.e., the higher the SD was, the lower E_comp_ and G moduli were, in agreement with the affine and phantom models for rubber elasticity and the literature data [9,85]. Therefore, for hydrogels within the same category, the E_comp_ (G) moduli followed the reverse order of the corresponding SDs, whose values conversely depended on the hydrogel crosslinking degree and the monomer concentration in the pre-hydrogel solution, and displayed a direct dependency on polymer hydrophilicity, as described in Section 2.2. Thus, E_comp_(G)_NVF10-XLG25_ > E_comp_(G)_NVF10-XLG15_, E_comp_(G)_NVF15-XLG20_ > E_comp_(G)_NVF15-XLG15_, and E_comp_(G)_NVF20-XLG15-MBA4_ > E_comp_(G)_NVF20-MBA4_ > E_comp_(G)_NVF20-MBA2_ > E_comp_(G)_NVF20-MBA1_ because of the decreasing crosslinking degree (increasing SD), E_comp_(G)_NVF20-XLG15_ > E_comp_(G)_NVF15-XLG15_ > E_comp_(G)_NVF10-XLG15_ due to the decreasing monomer concentration in the pre-gel solution (increasing SD), and E_comp_(G)_NVP10-XLG25_ > E_comp_(G)_NVF10-XLG25_ because of the increasing hydrophilicity of the polymer (increasing SD). One should mention that the doubly (physically and chemically) crosslinked structure of NVF_20_-XLG_15_-MBA_4_, by both clay and MBA crosslinks, led to a much higher E_comp_ value than that of the corresponding mono-crosslinked hydrogels, i.e., NVF_20_-XLG_15_ and NVF_20_-MBA_4_ (Table 1).

The calculated E/G ratio (E/G = 3.29–3.3, Table 1) was higher than the value expected for a rubbery material, i.e., E/G = 3, such as in swollen hydrogels. However, E/G ratios larger than 3 are usually obtained during the uniaxial compressive testing of hydrogels [86,87], depending on the strain range used to calculate G, as indicated by Equation (1) [87]:

G = E/(1 + 2λ^−3^) (1)

For extremely small deformations (λ → 1), an E/G value of 3 is obtained (Equation (1)). Therefore, the larger E/G ratios obtained in our case may be explained by the 0.98–0.93 deformation interval of linear strain–stress dependency we analyzed to determine G.

The ultimate compressive stress in the case of MBA-containing hydrogels, i.e., the fracture stress, increased with an increasing crosslinking density/decreasing SD, τ_c,max,NVF20-MBA1_ < τ_c,max_,_NVF20-MBA2_ < τ_c,max,NVF20-MBA4_ < τ_c,max_,_NVF20-XLG15-MBA4_, thus preserving the tendency noticed for E_comp_. The ultimate compressive strain was similar, i.e., within the 70–80% range, for these hydrogels, irrespective of their E_comp_ or τ_c,max_ (Table 1). As far as the NVF-XLG nanocomposite hydrogels were concerned, their τ_c,max_ values, as we defined here as the stress corresponding to 90% strain, correlated less well with the SDs of the hydrogels, most probably because 90% strain represented more of an arbitrary limit than a physically meaningful one.

The compressive toughness, which is a measure of a material’s resistance to destruction [88], depended also on the crosslinking density in the case of MBA-containing hydrogels, the T_c_ values increasing with this property of the hydrogel, similarly with τ_c,max_ and E_comp_, and decreasing with the SD of the hydrogel (Table 1). In the case of the NC hydrogels, the T_c_ values followed the same order as the ultimate compressive stress, which was different from that of the E_comp_ values (Table 1). Among all hydrogels, both nanocomposite and MBA-containing, by far the highest resistance to destruction was displayed by NVF_20_-XLG_15_-MBA_4_, probably because of its doubly crosslinked character.

The tensile mechanical tests were carried out at room temperature on hydrogel rods, swelled in DW for 11–13 days. Both the elastic modulus (E_tens_), the ultimate tensile stress (τ_t,max_) and the associated ultimate tensile strain (λ − 1)_max_, and the tensile toughness (T_t_) were determined as mean values of four-measurement sets (Table 2). The results showed the very good stretchability of the NVF-XLG nanocomposite hydrogels, in the 300–840% ultimate tensile strain range, unlike the MBA-containing NVF hydrogels, whose (λ − 1)_max_ was around 100% (Table 2, Figure 8c and Figure S8a–c). This difference may be ascribed to the dissimilar architecture of the two hydrogel network types, displaying a homogeneous distribution of the crosslinking sites in the case of the NVF-XLG nanocomposite hydrogels, which allowed to avoid stress concentration, and therefore larger material strains until failure were obtained. By contrast, the heterogeneous structure of the MBA-containing covalent hydrogels led to stress concentration on some of the covalent bonds, which broke at smaller deformations, finally determining the failure of the material at much lower strains than for the XLG-crosslinked hydrogels.

The behavior of the NVP_10_-XLG_25_ hydrogel, although crosslinked by XLG only, was similar to that of the MBA-crosslinked hydrogels, and different from that of NVF_10_-XLG_25_ (Table 2, Figure 8c and Figure S8c). This may be explained through the much bulkier pyrrolidone group attached to the hydrocarbon chain and the hydrophobic interactions occurring among the amphiphilic NVP monomer units. These factors lead to PNVP polymer segments much stiffer than in the case of PNVF, where the less bulky O=CH-NH- substituent of the hydrocarbon chain and the absence of extensive physical bonds among the NVF monomer units allow for a much higher flexibility in the polymer segments, which are thus able to adapt more easily to higher tensile stresses.

The explanations provided here may account also for the different behavior between the NVF-XLG hydrogels on one side and the MBA-crosslinked and NVP-XLG hydrogels on the other side during their compressive mechanical testing, i.e., no fracture up to at least 95% strain, but only hydrogel irreversible deformation at strains larger than 90%, in the former case, and the presence of a well-defined fracture within the 60–80% strain range in the latter (Table 1, Figure 8b).

The elastic moduli measured by tensile tests (E_tens_) reasonably agreed with the corresponding E_comp_ (Table 1, Table 2 and Table S1), as expected, confirming the accuracy of the experiments. Therefore, the E_tens_ moduli of the hydrogels belonging to the same category followed the same order as in the case of the E_comp_ ones, in agreement with the hydrogel crosslinking degree and the monomer concentration in the pre-hydrogel solution, as well as the polymer hydrophilicity: E_tens,NVF10-XLG25_ > E_tens,NVF10-XLG15_, E_tens,NVF15-XLG20_ > E_tens,NVF15-XLG15_, and E_tens,NVF20-XLG15-MBA4_ > E_tens,NVF20-MBA4_ > E_tens,NVF20-MBA2_ > E_tens,NVF20-MBA1_ because of the decreasing crosslinking degree, E_tens,NVF20-XLG15_ > E_tens,NVF15-XLG15_ > E_tens,NVF10-XLG15_ due to the decreasing monomer concentration in the pre-gel solution, and E_tens,NVP10-XLG25_ > E_tens,NVF10-XLG25_ because of the increasing hydrophilicity of the polymer.

The ultimate tensile stress, i.e., the stress corresponding to hydrogel fracture, increased with an increasing crosslinking density for both XLG-NVF hydrogels (τ_t,max,NVF10-XLG25_ > τ_t,max,NVF10-XLG15_; τ_t,max,NVF15-XLG20_ > τ_t,max,NVF15-XLG15_) and MBA-crosslinked ones (τ_t,max,NVF20-MBA4_ > τ_t,max,NVF20-MBA2_), as well as with increasing monomer concentration in the pre-gel solution (τ_max,NVF20-XLG15_ > τ_t,max,NVF15-XLG15_ > τ_t,max,NVF10-XLG15_). The largest τ_t,max_ among all hydrogels synthesized was displayed by NVF_20_-XLG_15_-MBA_4_ due to its double crosslinking by both XLG and MBA (Table 2, Figure 8c and Figure S8).

The tensile toughness, i.e., work to fracture, correlated closely with the ultimate tensile stress (fracture stress), with both XLG-NVF hydrogels and the MBA-crosslinked ones displaying the same order as a function of these tensile properties (Table 2).

In order to check the reproducibility of the compression and tensile values obtained, the confidence intervals for a 95% confidence level were calculated for the compressive and tensile mechanical properties of the hydrogels (Table S2). The results indicated good and very good reproducibility for the compression test data, except for the τ_c,max_ values of some NVF-XLG hydrogels, probably because they corresponded to an arbitrarily chosen ultimate strain value. As far as the tensile measurements are concerned, the calculated confidence intervals indicated a lower reproducibility for the measurements. This may be explained by the way how the testing hydrogel samples were obtained. While all the compression measurement specimens of a hydrogel were obtained from the same test tube, in the case of the tensile measurements, each specimen was obtained from a different tubing. Although the same pre-gel solution was used for the preparation of all specimens, the particular conditions for each tubing, such as remaining oxygen content or processing, may have been unintentionally slightly different, resulting in specimens with larger differences among their properties.

The synthesized hydrogels were further investigated by cyclic compression tests in order to get insight into their elastic character, mechanical stability, and shape-recovery ability, as a function of the crosslinking type, i.e., XLG- or MBA-promoted, and, therefore, to better understand the hydrogel network structure. A 50% maximum strain was typically applied in each test. The elastic character of a hydrogel was assessed by calculating the fraction of the dissipated energy out of the corresponding compression energy, (E_d_)_f_, during each compression cycle. The dissipated energy, i.e., the energy lost during the compression–relaxation cycle of the viscoelastic hydrogel, may be considered a measure for the viscous character of the material, including here damages to the hydrogel network structure, such as covalent/physical bond breakage [89,90]. The mechanical stability of the hydrogels, i.e., how constant the mechanical properties were, during repeated use was estimated based on the variation of the maximum stress (σ_max_) in the cycle as the compression cycle number increased. No or a very small change in σ_max_ during a cyclic compression test indicates a higher mechanical stability. The shape-recovery ability was assessed through the value of the residual strain λ_r_, which was the strain corresponding to zero stress of the unloading curve [90].

**Figure 9:**
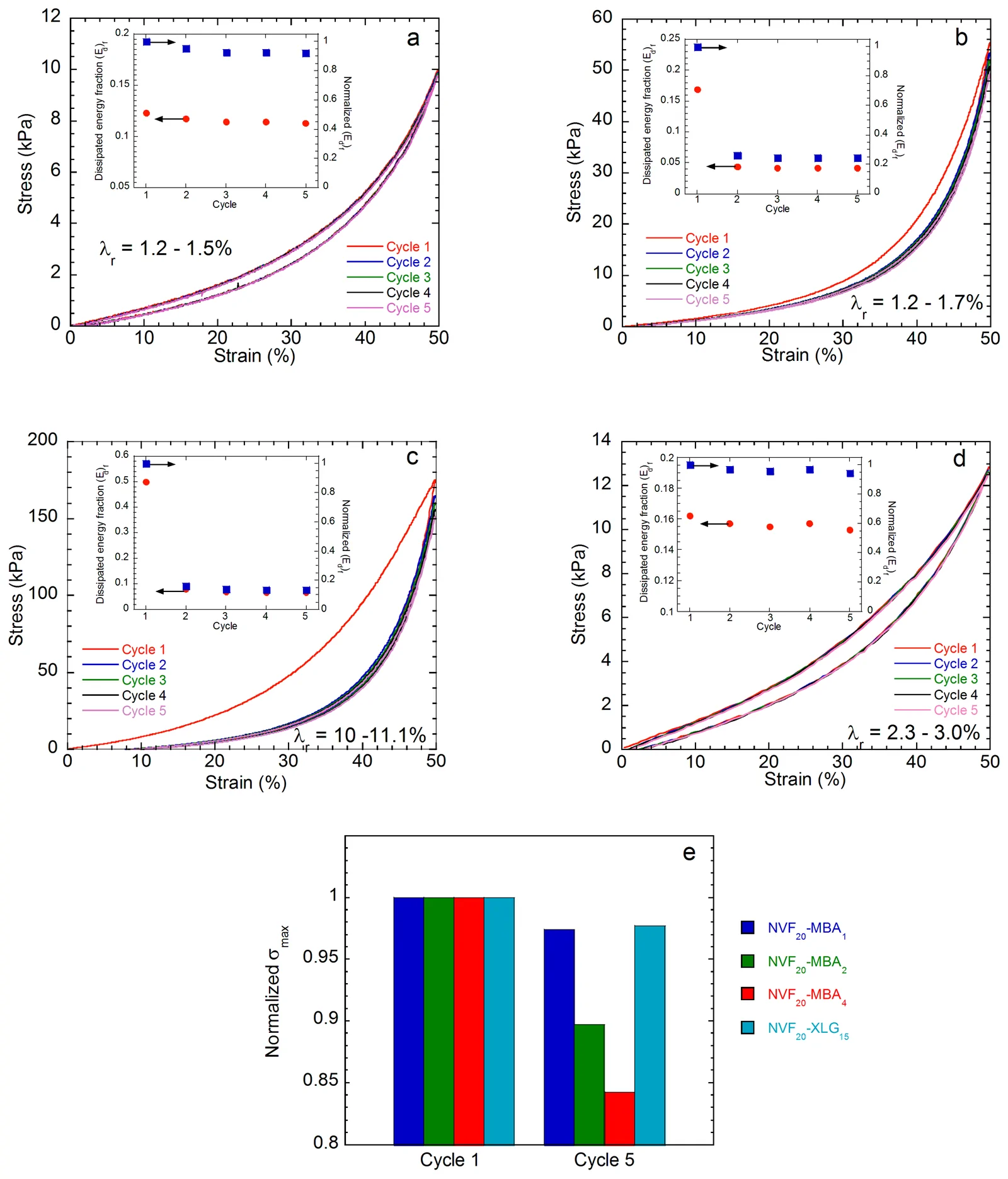
Cyclic compression tests: comparison between the properties of the MBA-crosslinked hydrogels and the XLG-crosslinked ones. Stress–strain curves for 5 successive loading–unloading cycles, the corresponding residual strain λ_r_ range, and the dissipated energy fraction (E_d_)_f_ (inset) in the case of (**a**) NVF_20_-MBA_1_, (**b**) NVF_20_-MBA_2_, (**c**) NVF_20_-MBA_4_, and (**d**) NVF_20_-XLG_15_. (**e**) The modification of the maximum stress (σ_max_) after 5 compression cycles for these hydrogels. Maximum strain = 50%.

The cyclic compression tests performed further confirmed the less homogeneous structure of the MBA-crosslinked hydrogels in comparison with that of the XLG-crosslinked ones. A large fraction of E_d_ was noticed for the NVF_20_-MBA_4_ first compression cycle, i.e., (E_d_)_f_ ≈ 0.5, which progressively decreased as the proportion of the crosslinker within the network became lower, i.e (E_d_)_f_ ≈ 0.17 in the case of NVF_20_-MBA_2_ and (E_d_)_f_ ≈ 0.12 for NVF_20_-MBA_1_ (Figure 9a–c). After a sharp decrease, starting with the second compression cycle, (E_d_)_f_ changed very little as the cycle number increased for both NVF_20_-MBA_4_ and NVF_20_-MBA_2_ (insets in Figure 9b,c). Unlike the hydrogels with higher MBA content, NVF_20_-MBA_1_ displayed a small and smooth (E_d_)_f_ decrease from the first to the fifth cycle (Figure 9a, inset). This behavior may be explained by the higher-MBA-content hydrogels possessing a more heterogeneous network, with a very broad distribution of the polymer chain length/molecular weight between two crosslinking points. As previously mentioned, the large reactivity difference between NVF and MBA led to more numerous short chains between the crosslinking sites as the MBA/NVF ratio increased. These short chains lacked the ability to accommodate higher mechanical loads, and therefore, they were the first covalent structures within the network that broke during the compression test. The hydrogel network resulting after the first compression cycle, i.e., after the breaking of most of the stress-concentrating chains, displayed a more homogeneous structure from this point of view, and therefore, a much smaller number of chains fractured during the second to the fifth cycle. This led to much lower (E_d_)_f_ values for the compression cycles no. 2–5 as compared to the first one, and also to a much smaller (E_d_)_f_ decrease as the cycle number increased (insets in Figure 9b,c). The results also showed a much lower (E_d_)_f_ during the first cycle in the case of NVF_20_-MBA_1_ than for NVF_20_-MBA_2_ and NVF_20_-MBA_4_ (Figure 9a–c), which may be explained by a hydrogel network that was more elastic, with a smaller number of stress-concentrating short chains between the crosslinking points because of the lower amount of MBA, as explained previously. However, as we will show later, this does not mean that the network of NVF_20_-MBA_1_ was more homogeneous. Also, one should keep in mind that other processes, besides the covalent bond breakage, such as temporary entanglements or other reversible molecular rearrangements, may have contributed to E_d_ as well, i.e., to the viscous character of a hydrogel. That is why hysteresis was still present during all five cycles in the case of NVF_20_-MBA_1_, as well as cycles no. 2–5 for NVF_20_-MBA_2_ and NVF_20_-MBA_4_.

The NVF_20_-XLG_15_ hydrogel displayed a low E_d_ fraction, which changed very little as the cycle number increased (Figure 9d), thus demonstrating the higher elastic character of the NVF NC gels and confirming the homogeneous structure of their network. This behavior, recorded at 50% maximum strain, was similar to that of NVF_20_-MBA_1_, but by increasing the maximum strain to 70%, i.e., raising the maximum stress applied, the differences between the network structures of these two hydrogels became obvious (Figure S9). Thus, a larger hysteresis difference was noticed between the first and the second cycle for NVF_20_-MBA_1_ (normalized (E_d_)_f_ = 0.77 for the second cycle), indicative of chain breaking processes occurring during the first compression cycle, unlike NVF_20_-XLG_15_, where the E_d_ fraction decrease (normalized (E_d_)_f_ = 0.95 for the second cycle) was small when looking at the same cycles (Figure S9a,b). These results support our statement above that the NVF_20_-MBA_1_ hydrogel network was still inhomogeneous, but with a chain-length-between-crosslinks distribution shifted toward higher molecular weights due to the lower MBA concentration, which determined extensive chain breaking starting to occur at higher stress values.

The shape-recovery ability of the hydrogels was good for almost all hydrogels discussed here, as indicated by residual strains (λ_r_) of a few percent (Figure 9a,b,d and Figure S9a,b), while higher residual strains of 10–11%, i.e., lower shape-recovery ability, were displayed by NVF_20_-MBA_4_, in agreement with its more rigid structure and larger network damages occurred during the compression cycles.

The mechanical stability of the MBA-crosslinked hydrogels became lower with an increasing MBA/NVF mole ratio, as can be seen from the normalized maximum stress (σ_max_) decreasing in the order NVF_20_-MBA_1_ > NVF_20_-MBA_2_ > NVF_20_-MBA_4_ in the case of compression cycle no. 5 (Figure 9e), as expected, taking into account the number of chains broken in the hydrogels. The mechanical stability of NVF_20_-MBA_2_ and NVF20-MBA_4_ was low, σ_max_ dropping to less than 90% in the fifth cycle in their case, while a high mechanical stability was displayed by NVF_20_-MBA_1_ (normalized σ_max_ ≈ 0.97) at 50% strain, similar to that of NVF_20_-XLG_15_ (Figure 9e). However, at 70% strain, the normalized σ_max_ was higher in the case of the NC hydrogel (0.92 for NVF_20_-XLG_15_ vs. 0.8 for NVF_20_-MBA_1_ after the fifth cycle), in agreement with its more homogeneous network (Figure S9c).

The behavior of NVF_20_-XLG_15_-MBA_4_ under cyclic compression was mostly determined by the presence of the covalent crosslinker within its network structure, i.e., a relatively large hysteresis during the first compression cycle as compared to the energy dissipated during the following ones, indicating extensive occurrence of chain breaking reactions (Figure S10a). The decrease in the normalized σ_max_ to 0.87 in the fifth cycle (Figure S10b) pointed out at the same conclusion as well.

**Figure 10:**
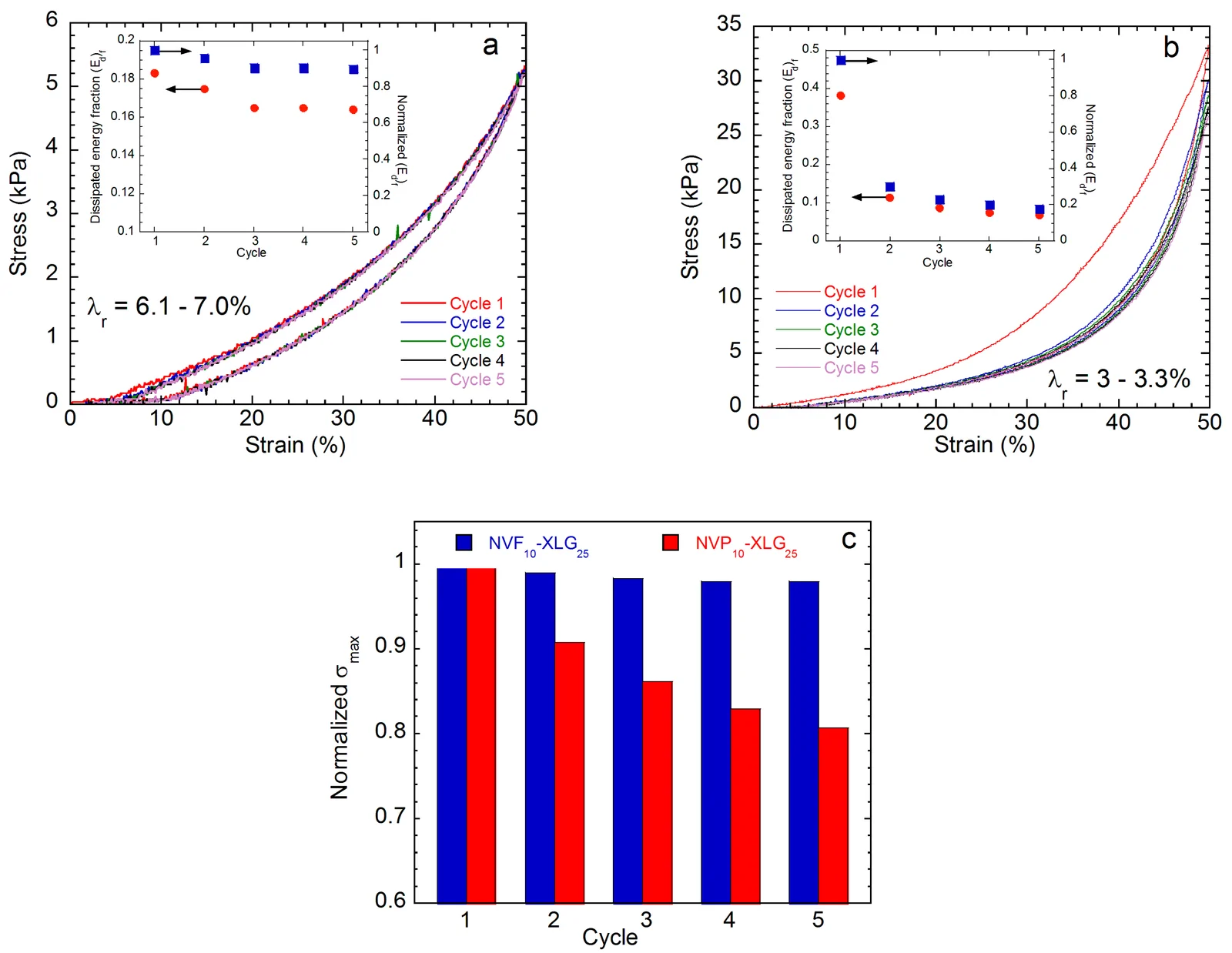
Cyclic compression tests: influence of the polymer nature—comparison between the properties of NVF_10_-XLG_25_ and NVP_10_-XLG_25_. Stress–strain curves for 5 successive loading–unloading cycles, the corresponding residual strain λ_r_ range, and the dissipated energy fraction (E_d_)_f_ (inset) in the case of (**a**) NVF_10_-XLG_25_ and (**b**) NVP_10_-XLG_25_. (**c**) The modification of the maximum stress (σ_max_) as a function of compression cycle. Maximum strain = 50%.

The cyclic compression tests also confirmed the differences between NVF_10_-XLG_25_ and NVP_10_-XLG_25_ hydrogels (Figure 10 and Figure S11), as revealed by the mechanical tests previously discussed (Figure 8b,c). Hence, although NVP_10_-XLG_25_ and NVF_10_-XLG_25_ were both NC hydrogels crosslinked only by XLG, the behavior of the first one during the cyclic compression tests was closer to that of the MBA-crosslinked hydrogels while the performance of the second one was similar to that of NVF_20_-XLG_15_, in agreement with the results of the compressive and tensile mechanical tests (Figure 8b,c). Thus, NVP_10_-XLG_25_ showed a lower elastic character, as indicated by the large E_d_ fraction of about 0.38 during the first compression cycle, which appreciably decreased during the second one (normalized (E_d_)_f_ = 0.3, Figure 10b), unlike NVF_10_-XLG_25_, in which case the E_d_ fraction during the first compression cycle was about 0.18 and the E_d_ fraction decrease from the first to the second compression cycle was very small (normalized (E_d_)_f_, second cycle = 0.96, Figure 10a). Also, the mechanical stability of NVP_10_-XLG_25_ proved to be much lower than that of NVF_10_-XLG_25_, as evidenced by the normalized σ_max_ values, significantly lower in the case of the NVP_10_-XLG_25_ hydrogel (Figure 10c).

These behavior differences between NVP_10_-XLG_25_ and NVF_10_-XLG_25_ under cyclic compression may be ascribed to the much bulkier pyrrolidone group attached to the hydrocarbon chain, and the presence of hydrophobic interactions among the NVP monomer units, as explained before in the case of compressive/tensile mechanical tests. The larger pyrrolidone groups as chain substituents lead to PNVP polymer segments much stiffer than in the case of PNVF, whose polymer chain, with less voluminous substituents, is more flexible and, therefore, able to adapt more easily to higher compressive stresses in order to avoid breaking, which does not happen for PNVP.

A comparison of the network homogeneity and mechanical properties between the NVF-XLG hydrogels reported within this work and covalently crosslinked PNVF hydrogels (Entries 1–6) and some Laponite-crosslinked NC hydrogels (Entries 7–10) reported in the literature is displayed in Table 3. In the absence of mechanical tests results, the homogeneity of the hydrogel network was estimated based on the mutual reactivity of the monomer and crosslinker in the copolymerization reaction. A similar reactivity leads to a homogeneous network while a very different one leads to a heterogeneous network. Thus, the crosslinking monomers with *N*-vinylformamide polymerizable groups (NVEE, BDEP) are expected to display reactivities very close to that of NVF because of the identical structure of the polymerizable moiety. The same is expected for BVU, whose polymerizable groups possess a very similar chemical structure with that of NVF. The vinyl polymerizable group of PEGDA, of the acrylic type, i.e., a conjugated one, is more reactive than the unconjugated double bond of NVF. Therefore, their crosslinking copolymerization leads to heterogeneous hydrogel networks. The same for the NVF crosslinking polymerization initiated by heat, where the network formation is based on free radical chain transfer and termination by combination reactions.

To the best of our knowledge, ref [9] (entry 1) is the only one in the literature reporting the mechanical properties of covalently crosslinked PNVF hydrogels. The results of the compressive mechanical tests upon the hydrogels described within this work were in the range with the compression tests values obtained for our hydrogels (entry 11), except E_comp_, which reached larger values in the case of NVF-NVEE hydrogels. One should also mention that the behavior of the NVF-NVEE hydrogels during the compression testing was different from that of the NVF-XLG hydrogels. Namely, the NVF-NVEE hydrogels broke at the ultimate compressive strength value during the mechanical testing [9], while the NVF-XLG hydrogels did not do that during the compression testing, but only deformed instead (Figure S7). No tensile tests were provided for the NVF-NVEE hydrogels.

Table 3 also displays a comparison between the mechanical properties of the NVF-XLG NC hydrogels synthesized within this work and NC hydrogels with different monomers described in the literature. We could not find compression testing data for NC hydrogels in the literature, but only found results of the tensile tests. According to Table 3, the NVF-XLG NC (entry 11) possessed similar E_tens_ values with all the other NC hydrogels listed (entries 7–10) but lower ultimate tensile strength. As far as stretchability was concerned, our hydrogels displayed a ultimate tensile strain similar to that of the polyDMAEMA hydrogel but appreciably lower than in the case of the NC hydrogels with a polyNIPAM-, polyAAm-, or polyDMAA-based network.

## 3. Conclusions

Novel highly compressible and stretchable nanocomposite (NC) hydrogels were successfully synthesized by the polymerization of NVF in aqueous solution in the presence of Laponite XLG as the only crosslinker and AIBN as the thermal initiator, and their expected composition was demonstrated by FTIR, TEM, XRD, and TGA analyses.

Swelling degree and mechanical measurements showed that the properties of the PNVF NC hydrogels were largely different from those of both the PNVF hydrogels covalently crosslinked by MBA and equivalent PNVP nanocomposite hydrogels crosslinked by XLG. Regarding the water-swelling behavior, the NVF-XLG hydrogels displayed a slow, but steady, SD increase with time after an initial fast swelling stage, unlike the MBA-crosslinked and NVP-XLG hydrogels, which, after a similar fast swelling first stage, showed a much smaller change in the swelling degree that either decreased in the first case or increased in the second one. Attempts to prove that this continuous SD increase with time in the case of NVF-XLG hydrogels was due to the sluggish decrease in the hydrogel crosslinking density as a consequence of the gradual degradation/dissolution of XLG in DW and/or the slow hydrolysis of the NVF units failed to indisputably support this hypothesis.

The mechanical testing of the synthesized hydrogels by uniaxial compressive and tensile measurements showed the much higher compressibility and stretchability of the NVF-XLG NC hydrogels in comparison with both NVP-XLG and MBA-crosslinked NVF hydrogels. This difference was explained through the more homogeneous hydrogel network of the NVF-XLG hydrogels, unlike the MBA-crosslinked NVF ones, and by the more flexible NVF polymer segments in comparison with the NVP ones. A doubly crosslinked NVF hydrogel, i.e., by both XLG and MBA crosslinking agents, displayed mechanical properties dominated by the MBA content, i.e., a larger elastic modulus, ultimate strength, and toughness than the corresponding mono-crosslinked hydrogels because of the higher crosslinking density, but a relatively low compressibility and stretchability in comparison with the corresponding XLG-only-crosslinked hydrogel.

The conclusions of the compressive and tensile measurements were supported by the results of the cyclic compression tests. Large hysteresis values for the first compression cycle in comparison with the second one, indicative for a lower elastic character, were recorded in the case of MBA-crosslinked hydrogels, unlike the NVF-XLG ones. This was ascribed to the less homogeneous network, with many short chains between the crosslinking points, which broke at smaller deformations due to the stress concentration on them. The same behavior was noticed for the NVP-XLG nanocomposite hydrogel, which was explained through the PNVP segments being much stiffer than in the case of PNVF due to the bulky pyrrolidone substituent groups and the hydrophobic interactions occurring among the amphiphilic NVP monomer units. This made the PNVP segments unable to adapt fast enough to higher compressive stresses, which led to chain breakage.

The cyclic compression tests also revealed a lower mechanical stability, i.e., a more pronounced decrease in the σ_max_ values, under repeated loading–unloading cycles of both MBA-crosslinked and PNVP hydrogels as compared to the PNVF NC gels.

A comparison of the compressive mechanical properties of our hydrogels with those of a homogeneous-network, covalently crosslinked NVF-NVEE hydrogel showed in-range values for ultimate compressive strength and ultimate strain while E_comp_ reached larger values in the case of NVF-NVEE hydrogels. Also, by comparing the mechanical properties of the NVF-XLG NC hydrogels synthesized within this work and NC hydrogels with different monomers described in the literature, it resulted that our hydrogels possessed similar E_tens_ values with other NC hydrogels having DMAEMA, NIPAM, AAm and DMAA as monomers but lower ultimate tensile strength. As far as stretchability was concerned, our hydrogels displayed an ultimate tensile strain similar to that of the polyDMAEMA hydrogel but appreciably lower than in the case of the NC hydrogels with polyNIPAM, polyAAm, or polyDMAA networks.

## 4. Materials and Methods

### 4.1. Materials

*N*-Vinylformamide (NVF, Sigma-Aldrich, Steinheim, Germany, 98%) and *N*-vinyl-2-pyrrolidone (NVP, ACROS Organics, Geel, Belgium, 98%) were distilled under vacuum and stored in a freezer. 2,2′-Azobis(2-methylpropionitrile) (AIBN, Fluka, Buchs, Switzerland, 98%) was recrystallized from methanol and kept in a refrigerator at 4 °C. Laponite XLG (XLG, BYK Additives & Instruments, kindly provided by Cosichem & Analytical, Bucharest, Romania), *N*,*N*’-methylenebisacrylamide (MBA, Merck, Hohenbrunn, Germany, 98%), and isopropanol (Chimreactiv S.R.L.,Bucharest, Romania, 99.9%) were used without further purification. Distilled water (DW) was employed in all experiments employing water.

### 4.2. Hydrogel Preparation

To prepare the nanocomposite hydrogels, appropriate amounts of DW and XLG (Table 4) were added to a 25 mL Schlenk flask and magnetically stirred at room temperature for 2 h to exfoliate the clay, followed by the addition of MBA, if it was the case. The mixture was further stirred until full dissolution of MBA. In the case of the MBA-only-crosslinked hydrogels, this first step of the hydrogel synthesis procedure was the same but skipped XLG addition. Separately, an AIBN (0.25 mol% to NVF) solution in NVF (10 wt%, 15 wt% or 20 wt% to the whole polymerization mass) was prepared, which was then added to the Schlenk flask, and the polymerization mixture was additionally stirred for 15 min. The resulting homogeneous solution/dispersion was degassed by cycling it 5 times between the vacuum obtained from a water vacuum pump and nitrogen and transferred via purged syringes into either nitrogen-purged, rubber septum-sealed glass test tubes (9 mm inner diameter), in the case of the compression mechanical tests, or nitrogen-purged 100 mm length glass tubings (4 mm inner diameter) sealed by a rubber septum at each end, in the case of tensile mechanical tests. The test vials and the tubings were then placed in an oil bath or in an oven, respectively, at 50 °C for 24 h.

At the end of the heating time, both test tubes and tubings were cooled down and further processed in different ways. The test tubes were carefully broken, and the resulting hydrogel cylinders were cut into about 9 mm height pieces to be employed for the compression mechanical tests. Also, about 2 mm thick disk-shaped hydrogel pieces were obtained for conversion (C), gel fraction (GF), and swelling degree (SD) determinations. The hydrogel cylinders were purified by immersing them in DW for 11–13 days, during which interval water was replaced 8 times. Then, some of them were employed for compression mechanical tests while some others were used to determine the SD values of the samples at the mechanical testing time. To determine C and GF, a certain number of as-prepared disk-shaped hydrogels (W_h,0_) were first thoroughly dried, initially in atmosphere and then in a desiccator over anhydrous CaCl_2_, to constant weight (W_x_), followed by purification in DW as described above. The resulting purified water-swollen hydrogels were dried, first in an oven at 50 °C for a few days and then in an anhydrous CaCl_2_-containing desiccator to constant weight (W_x,f_). The monomer conversion was calculated by means of Equation (2) while GF was calculated by employing Equation (3) [60].

C(%) = (W_x_ − W_h,0_ × w_xlg_)/(W_h,0_ × w_monomer_) × 100 (2)

where w_xlg_ and w_monomer_ were the mass fractions of XLG and monomer, respectively, in the pre-hydrogel solution.

GF(%) = W_x,f_/W_x,0_ × 100 (3)

The glass tubings were opened by removing the rubber septa, and those containing the poly(*N*-vinylformamide) (PNVF) hydrogels were immersed into 50 mL centrifuge tubes filled with isopropanol. After several days, the hydrogels shrank inside the tubings due to the precipitation of PNVF, and the white polymer rod thus formed was obtained in some cases by simply extracting it from the tubing or by breaking the tubing in some others. The poly(*N*-vinyl-2-pyrrolidone) (PNVP) hydrogels were easily removed from the tubings by pushing them out. Both PNVF xerogels and PNVP hydrogels were purified by immersing them in DW for 11–13 days, during which interval water was replaced 8 times. Then, the swollen flexible hydrogel rods were cut at about 9 cm length and these pieces were used for the tensile mechanical measurements while the remaining hydrogel pieces were employed to determine the SD values of the samples at the mechanical testing time.

### 4.3. Swelling Degree Determinations

To determine the SD values of the hydrogels used for the mechanical (compression, tensile) analyses, the purified and swollen hydrogel pieces were removed from water, superficially wiped with moisturized filter paper, and weighed (W_h_). Next, the hydrogels were dried first in an oven at 50 °C for about 5–7 days and then in a desiccator over anhydrous CaCl_2_ to constant weight (W_x_). The SD was calculated according to Equation (4) and reported as the average value ± standard deviation of the SDs recorded for 3 equivalent hydrogel samples.

SD = W_h_/W_x_ (g hydrogel/g xerogel) (4)

The SD vs. time experiments were carried out at 25 ± 1 °C in a chilling/heating dry plate (Torrey Pines Scientific Inc., Carlsbad, CA, USA) by using purified and fully dried xerogel disks, which had previously been polished to reach 1 ± 0.05 mm thickness. The weighed (W_x_) xerogel disk was sunk into 35 mL DW contained by a 50 mL centrifuge tube, and the swollen disk was removed from the tube at certain time intervals, wiped superficially with moisturized filter paper, weighed (W_h_), and placed back into water. The swelling degrees at various time intervals were calculated by using Equation (4) and reported as the average value ± standard deviation values of the SDs recorded for 3 equivalent hydrogel samples.

### 4.4. Characterizations

Attenuated Total Reflectance–Fourier Transform Infrared (FTIR) spectra were recorded on a Bruker Vertex 70 equipment (Bruker, Billerica, MA, USA) with a Bruker Platinum ATR modulus, in the 4000–400 cm^−1^ wavenumber range, with a resolution of 4 cm^−1^ and accumulation of 32 scans. Thermogravimetric (TGA) analyses were carried out on a NETZSCH TG 209F1 Libra instrument (Netzsch, Selb, Germany) by heating powdered xerogel samples of about 2 mg from room temperature to 700 °C. A 10 °C heating rate was applied while the sample was kept under nitrogen flow during the measurement.

X-ray diffraction (XRD) measurements were performed on a Rigaku Smartlab diffractometer (Rigaku Corporation, Tokyo, Japan) by using a Cu Kα (λ = 0.1541 nm) source. The diffractograms were recorded in parallel beam geometry over a 2θ range from 2° to 80° at a scanning speed of 2 °/min and a step size of 0.02°.

The transmission electron microscopy (TEM) images were obtained by means of G2 F20 TWIN Tecnai (FEI Company, Eindhoven, the Netherlands) equipment by analyzing nanocomposite xerogel powders.

The hydrogels were mechanically characterized by both unconfined uniaxial compression and tensile tests. The measurements were carried out on an Instron 3382 instrument (Instron, Norwood, MA, USA) equipped with a 2 kN cell at room temperature. Six or seven specimens were measured for each hydrogel, from which four specimens were selected according to the following criteria: the ones with the closest compressive moduli in the case of compression testing, respectively the closest ultimate tensile strains, in the case of tensile measurements. Based on the selected samples, the mean ± standard deviation and the confidence interval for a 95% confidence level was calculated and reported for each hydrogel. Hydrogel cylinders with the height of 8.2–13.6 mm and a diameter of 11.0–18.3 mm (depending on the swelling degree of the hydrogel) were employed for uniaxial compressive tests, at a 2 mm/min compression rate, until failure in the case of covalently crosslinked hydrogels or until about 90% strain in the case of nanocomposite hydrogels. Both elastic (Young’s, E) and compression (shear, G) moduli were determined from the stress–strain curves by applying Equations (5) and (6) to small strains (1 − λ) within the 2–7% range (linear plot region) [86]:

τ = E (1 − λ)(5)

τ = G (λ^−2^ − λ) (6)

where τ represents the stress while λ is the ratio between the instantaneous (l) and initial (l_0_) height of the cylindrical hydrogel specimen. Also, the fragmentation stress, i.e., the stress value right before the hydrogel failure occurred, in the case of the covalently crosslinked hydrogels, and the stress corresponding to 90% strain, in the case of nanocomposite hydrogels, as well as the corresponding strain, were reported as the ultimate compressive stress (compression strength, τ_c,max_) and ultimate compressive strain (1 − λ)_max_, respectively. The compressive toughness (T_c_) of the hydrogels, i.e., the work to fracture, in the case of covalently crosslinked hydrogels, or to 90% strain, in the case of nanocomposite hydrogels, was calculated as the area under the stress–strain curve up to these points [89,91]. The specimens used for the tensile mechanical tests were hydrogel cylinders (rods) of about 90 mm length and diameters of 5.3–7.4 mm (depending on the swelling degree of the sample). The hydrogels were fixed between clamps having sandpaper faces, resulting in an effective length (l_0_) of 30.3–41.3 mm after applying a preload of 0.1 N. The tensile measurements were carried out at a crosshead speed of 100 mm/min until specimen failure. The elastic modulus was determined from the stress–strain curves by applying Equation (7) to small strains (λ − 1) within the 2–7% range, where the plots were linear.

τ = E (λ − 1)(7)

Cyclic compression measurements were performed on a CT3 Texture analyzer (Brookfield Engineering Laboratories Inc., Middleboro, MA, USA) equipped with a 4500 g load cell and a TA4/1000 compression accessory. Cylindrical hydrogel samples, similar to those used for the uniaxial compression tests, were employed. The hydrogels were placed on the bottom plate of the instrument and 5 successive loading–unloading cycles without a waiting time, at a crosshead speed of 0.5 mm/s, were performed on each specimen. A 50% maximum strain was typically applied in each test. The dissipated (absorbed) energy (E_d_, J/m^3^) was calculated as the area inside the loading–unloading hysteresis loop, i.e., the difference between the area under the loading curve and that under the unloading one. The area beneath the loading curve represented the compression energy that was applied to the hydrogel during compression (J/m^3^) while the area under the unloading curve signified the relaxation energy (J/m^3^) [90].

## Figures and Tables

**Table 1:** The compressive mechanical properties of the synthesized hydrogels.

Sample	SD ^a^ g Hydrogel/g Xerogel	E_comp_ ^b^ kPa	G ^c^ kPa	E_comp_/G	τ_c,max_ ^d^ kPa	(1 − λ)_max_ ^e^ %	T_c_ ^f^ kJ/m^3^
NVP_10_-XLG_25_	22.3 ± 0.5	13.9 ± 0.2	4.20 ± 0.08	3.30	72.2 ± 11.5	62.9 ± 2.3	11.0 ± 1.5
NVF_10_-XLG_25_	34.0 ± 1.2	4.3 ± 0.1	1.32 ± 0.04	3.29	197.9 ± 42.1	90	15.4 ± 2.4
NVF_10_-XLG_15_	57.9 ± 3.6	1.2 ± 0.1	0.36 ± 0.03	3.30	675.6 ± 34.5	90	20.5 ± 1.4
NVF_15_-XLG_15_	33.1 ± 0.2	5.6 ± 0.1	1.70 ± 0.03	3.29	3765.7 ± 1822.1	90	97.7 ± 34.9
NVF_20_-XLG_15_	24.1 ± 0.2	13.8 ± 0.3	4.20 ± 0.10	3.29	4552.1 ± 1547.6	90	153.1 ± 42.5
NVF_15_-XLG_20_	24.6 ± 1.1	13.4 ± 1.0	4.07 ± 0.29	3.30	2189.37 ± 780.5	90	93.2 ± 22.2
NVF_20_-XLG_15_-MBA_4_	7.6 ± 0.3	338.2 ± 9.5	102.6 ± 2.9	3.30	3366.4 ± 527.6	72.9 ± 4.2	620.4 ± 129.3
NVF_20_-MBA_4_	15.1 ± 0.1	70.9 ± 1.1	21.50 ± 0.34	3.30	790.8 ± 64.5	77.0 ± 0.9	116.1 ± 8.4
NVF_20_-MBA_2_	28.2 ± 0.1	14.4 ± 0.3	4.37 ± 0.08	3.30	690.2 ± 113.6	78.5 ± 1.8	69.5 ± 10.1
NVF_20_-MBA_1_	40.0 ± 0.1	5.7 ± 0.1	1.73 ± 0.04	3.29	163.1 ± 20.7	77.3 ± 0.5	11.2 ± 1.2

^a^ Swelling degree of the hydrogel at the time of compression mechanical testing, i.e., after immersion in DW for 11–13 days. ^b^ Elastic modulus of the hydrogel determined by compression mechanical tests. ^c^ Compression (shear) modulus of the hydrogels synthesized. ^d^ Ultimate compressive strength, in the case of MBA-containing hydrogels and NVP_10_-XLG_25_ hydrogel, or the stress corresponding to 90% strain for the NVF-XLG nanocomposite hydrogels. ^e^ Ultimate compressive strain, in the case of MBA-containing hydrogels and NVP-XLG hydrogel, or 90%, in the case of NVF-XLG nanocomposite hydrogels. ^f^ Compressive toughness, calculated up to material failure, in the case of MBA-containing hydrogels and NVP-XLG hydrogel, or up to 90% strain for the NVF-XLG nanocomposite hydrogels.

**Table 2:** The tensile mechanical properties of the synthesized hydrogels.

Sample	SD ^a^ g Hydrogel/g Xerogel	E_tens_ ^b^ kPa	τ_t,max_ ^c^ kPa	(λ − 1)_max_ ^d^ %	T_t_ ^e^ kJ/m^3^
NVP_10_-XLG_25_	21.8 ± 0.1	12.3 ± 4.0	10.0 ± 1.1	80.8 ± 11.3	3.9 ± 0.8
NVF_10_-XLG_25_	35.1 ± 0.9	5.0 ± 2.5	23.3 ± 3.0	610.1 ± 69.4	74.2 ± 14.8
NVF_10_-XLG_15_	58.9 ± 1.0	2.3 ± 0.8	4.0 ± 0.3	299.2 ± 21.8	6.1 ± 0.9
NVF_15_-XLG_15_	37.8 ± 0.9	4.8 ± 1.6	23.4 ± 3.6	630.0 ± 75.2	70.6 ± 18.8
NVF_20_-XLG_15_	28.7 ± 0.5	9.0 ± 2.5	39.8 ± 16.1	560.1 ± 169.3	114.0 ± 67.8
NVF_15_-XLG_20_	26.7 ± 0.3	10.3 ± 1.0	56.4 ± 9.0	843.7 ± 207.7	252.9 ± 124.3
NVF_20_-XLG_15_-MBA_4_	8.0 ± 0.1	288.5 ± 9.2	440.1 ± 42.9	85.5 ± 9.6	179.7 ± 36.9
NVF_20_-MBA_4_	13.9 ± 0.1	62.4 ± 5.1	63.8 ± 9.0	113.7 ± 30.7	44.0 ± 17.9
NVF_20_-MBA_2_	27.6 ± 0.1	13.0 ± 2.0	31.6 ± 4.4	94.8 ± 8.5	10.8 ± 2.5

^a^ Swelling degree of the hydrogel at the time of tensile mechanical testing, i.e., after immersion in DW for 11–13 days. ^b^ Elastic modulus of the hydrogel determined by tensile mechanical tests. ^c^ Ultimate tensile strength. ^d^ Ultimate tensile strain. ^e^ Tensile toughness.

**Table 3:** Comparison of the network homogeneity and mechanical properties between the NVF-XLG hydrogels reported within this work and covalently crosslinked PNVF hydrogels and some Laponite-crosslinked NC hydrogels reported in literature.

Entry	Monomer	Crosslinking ^a^ Agent	Network Homogeneity ^b^	Compression			Tensile			Ref.
				E_comp_ ^c^ kPa	τ_c,max_ ^d^ kPa	(1 − λ)_max_ ^e^ %	E_tens_ ^f^ kPa	τ_t,max_ ^g^ kPa	(λ − 1)_max_ ^h^ %	
1	NVF	NVEE	homogeneous	3.4–90.7	303–1139	70–98	NR	NR	NR	[9]
2	NVF	heat	heterogeneous	NR	NR	NR	NR	NR	NR	[69]
3	NVF	BVU	homogeneous	NR	NR	NR	NR	NR	NR	[66]
4	NVF	PEGDA	heterogeneous	NR	NR	NR	NR	NR	NR	[11]
5	NVF	BDEP	homogeneous nanogels	NR	NR	NR	NR	NR	NR	[68]
6	NVF	NVEE	homogeneous microgels	NR	NR	NR	NR	NR	NR	[3]
7	DMAEMA	XLS	homogeneous	NR	NR	NR	0.4–1.9	52–282	243–659	[57]
8	NIPAM	XLG	homogeneous	NR	NR	NR	1.5–9.9	41–109	857–1424	[47]
9	AAm	XLS	homogeneous	NR	NR	NR	3.8–19.1	107–319	1672–2829	[49]
10	DMAA	XLG	homogeneous	NR	NR	NR	1.2–15.5	34.5–255.6	1264–1654	[52]
11	NVF	XLG	homogeneous	1.2–13.8	198–4552	≥90	2.3–10.3	4.0–56.4	299–843	present work

^a^ NVEE = 2-(*N*-vinylformamido)ethylether; BVU = 1,3-bisvinylethylene urea; PEGDA = poly(ethylene glycol) diacrylate, MW = 575; BDEP = 2-bis[2,2′-di(*N*-vinylformamido)ethoxy]propane; XLS = Laponite XLS; XLG = Laponite XLG. ^b^ In the absence of the mechanical tests, the homogeneity of the hydrogel network was estimated based on the mutual reactivity of the monomer and crosslinker in the copolymerization reaction. A similar reactivity leads to a homogeneous network while a very different one leads to a heterogeneous network. ^c^ Elastic modulus of the hydrogel determined by compression mechanical tests. ^d^ Ultimate compressive strength. In the case of the NVF-XLG nanocomposite hydrogels (present work), it is the stress corresponding to 90% strain. ^e^ Ultimate compressive strain: at least 90% in the case of NVF-XLG nanocomposite hydrogels. ^f^ Elastic modulus of the hydrogel determined by tensile mechanical tests. ^g^ Ultimate tensile strength. ^h^ Ultimate tensile strain. NR = not reported.

**Table 4:** Hydrogel samples prepared: compositions of the precursor mixtures ^1^ and gel fractions (GFs).

Hydrogel Sample ^2^	NVF ^3^ (g)	NVP ^4^ (g)	XLG ^5^ (g)	MBA ^6^ (g)	AIBN ^7^ (g)	DW (g)	GF (%)
NVF_10_-XLG_25_	1.0	-	0.250	-	0.006	8.75	91
NVP_10_-XLG_25_	-	1.0	0.250	-	0.006	8.75	87.5
NVF_10_-XLG_15_	1.0	-	0.150	-	0.006	8.85	85
NVF_15_-XLG_15_	1.5	-	0.225	-	0.009	8.20	91
NVF_20_-XLG_15_	2.0	-	0.300	-	0.012	7.70	93.5
NVF_15_-XLG_5_	1.5	-	0.075	-	0.009	8.40	71.5
NVF_15_-XLG_20_	1.5	-	0.300	-	0.009	8.20	91.5
NVF_20_-XLG_15_-MBA_4_	2.0	-	0.300	0.173	0.012	7.55	94
NVF_20_-MBA_4_	2.0	-	-	0.173	0.012	7.85	81.5
NVF_20_-MBA_2_	2.0	-	-	0.087	0.012	7.90	80
NVF_20_-MBA_1_	2.0	-	-	0.044	0.012	7.85	80

^1^ For 10 g of polymerization mixture. ^2^ The name of each sample contains the abbreviations of both monomer and crosslinking agent/agents and some numbers as subscripts indicating their proportion in the hydrogel precursor mixture. For example, NVF_20_-XLG_15_-MBA_4_ denotes the hydrogel obtained from NVF (20 wt% to the whole polymerization mixture) as the monomer by using both XLG (15 wt% to NVF) and MBA (4 mol% to NVF) as crosslinkers. ^3^ 10, 15 or 20 wt% based on the polymerization mixture. ^4^ 10 wt% based on the polymerization mixture. ^5^ 5, 15, 20, or 25 wt% based on monomer. ^6^ 1, 2, or 4 mol% based on NVF. ^7^ 0.25 mol% based on monomer.

## Data Availability

The data presented in this study are available on request from the corresponding author.

## Supplementary Materials

The following supporting information can be downloaded at: https://www.mdpi.com/article/10.3390/gels12010031/s1, Figure S1. FTIR spectra of the hydrogels synthesized. Figure S2. Comparison of the XRD patterns of Laponite XLG, some of the NC hydrogels synthesized, and the PNVF matrix. Figure S3. Dependence of the swelling degree of the hydrogels synthesized on hydrogel crosslinking degree, monomer concentration in the pre-hydrogel solution, and hydrogel monomer unit structure/properties. Figure S4. FTIR spectra of hydrogels stored in DW for more than one year in comparison with those of the corresponding hydrogels kept in DW for 11–13 days. Figure S5. The thickness of the synthesized hydrogels after swelling in DW, at room temperature, for 7 days. Figure S6. Typical compressive stress–strain curves for the hydrogels synthesized. Figure S7. The shape of NVF_15_-XLG_20_ nanocomposite hydrogel samples compressed at various strains. Figure S8. Typical tensile stress–strain curves for the hydrogels synthesized. Figure S9. Cyclic compression tests: stress–strain curves for 70% maximum strain: comparison between the properties of NVF20-MBA1 and NVF20-XLG15. Figure S10. Cyclic compression tests in the case of NVF20-XLG15-MBA4. Figure S11. Cyclic compression tests: comparison between NVP10-XLG25 and NVF10-XLG25. Stress–strain curves for 5 successive loading–unloading cycles. Table S1. Comparison of the elastic moduli obtained by compression (E_comp_) and tensile (E_tens_) tests. Table S2. Confidence intervals (95% confidence level) calculated for the compressive and tensile mechanical properties of the hydrogels synthesized.
