# Supplementary material for: Poly(N-vinyl formaldehyde)—Laponite XLG Nanocomposite Hydrogels: Synthesis and Characterization
Source: Gels. 2025 Dec 30;12(1):31. doi: 10.3390/gels12010031 (PMC12841149; doi:10.3390/gels12010031)
Supplement: Supplementary file 1 [file gels-12-00031-s001.zip › gels-4056942-supplementary.pdf]

Supplementary Data

Poly(N-vinyl formaldehyde) - Laponite XLG nanocomposite hydrogels: synthesis and characterization

Paul Octavian Stănescu[1,2], Andrada Serafim[1,2], Anita-Laura Chiriac[3], Anamaria Zaharia[3], Raluca Şomoghi[3,4], Mircea Teodorescu[1,*]

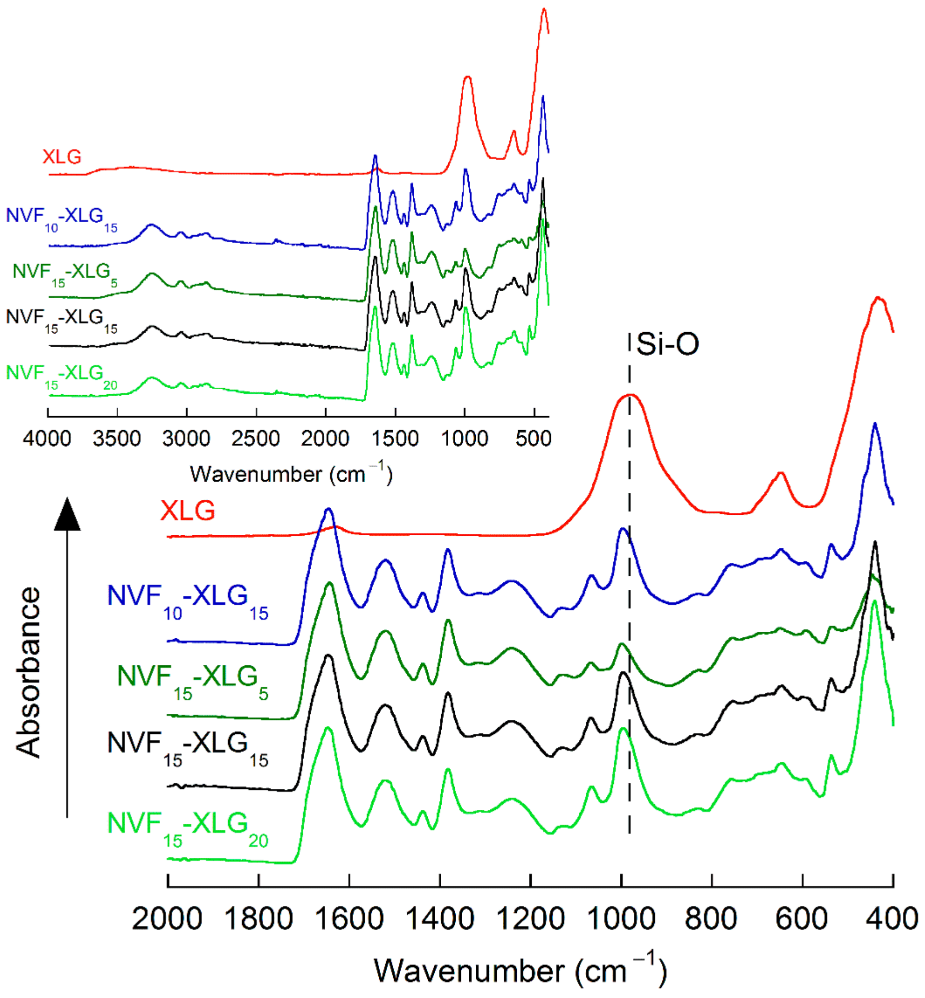

Figure S1. FTIR spectra of the hydrogels synthesized. The xerogel spectra were normalized against the band at 1647 cm^{-1}.

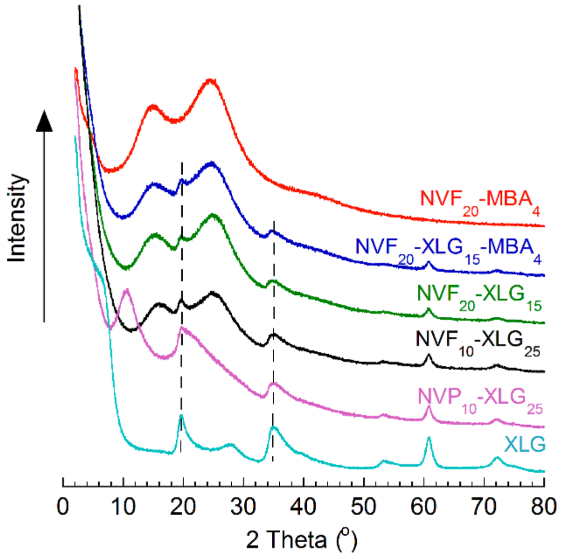

Figure S2. Comparison of the XRD patterns of Laponite XLG, some of the NC hydrogels synthesized and the PNVF matrix.

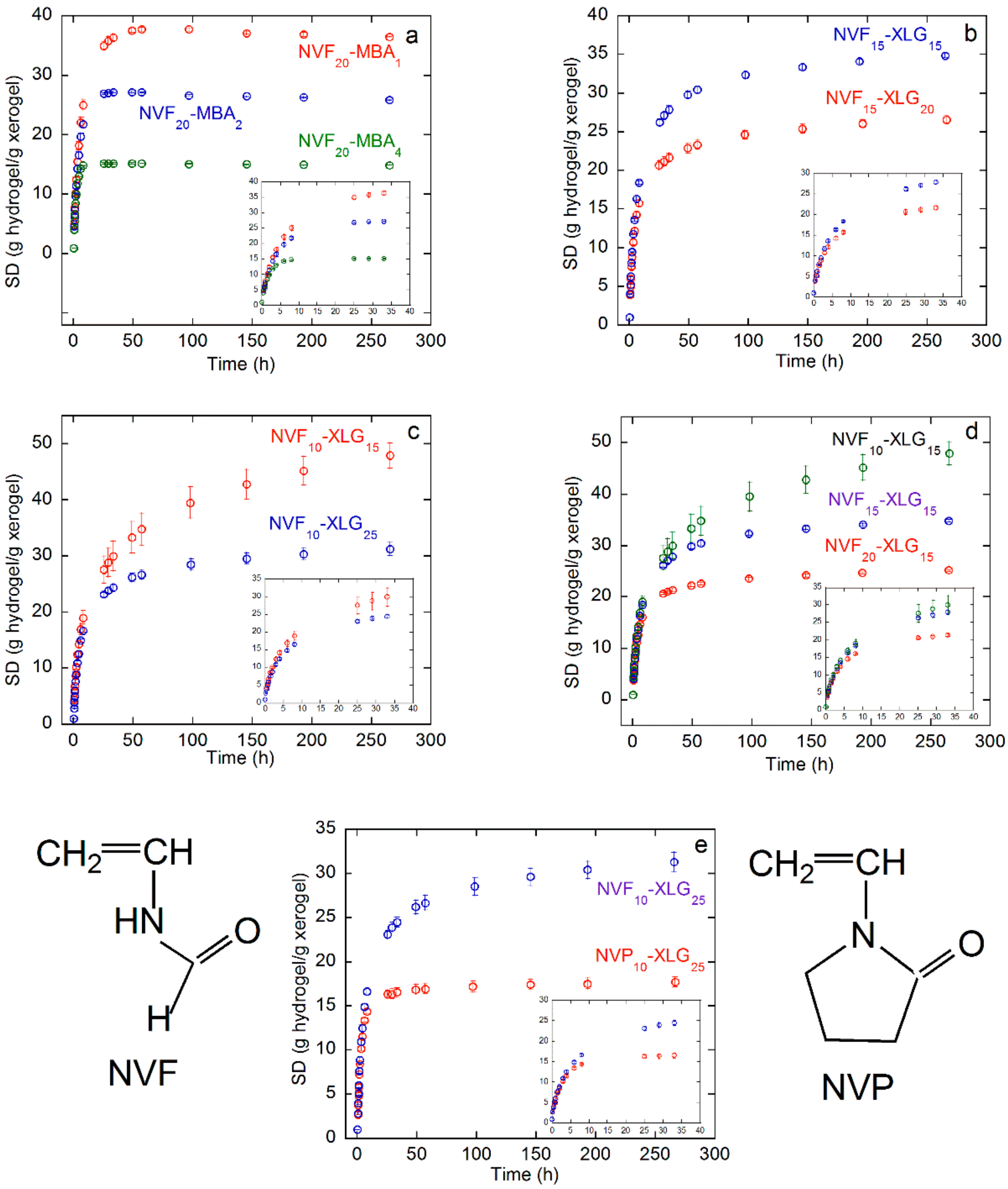

Figure S3. Dependence of the swelling degree of the hydrogels synthesized on hydrogel crosslinking degree (a, b, c), monomer concentration in the pre-hydrogel solution (d), and hydrogel monomer unit structure/properties (e).

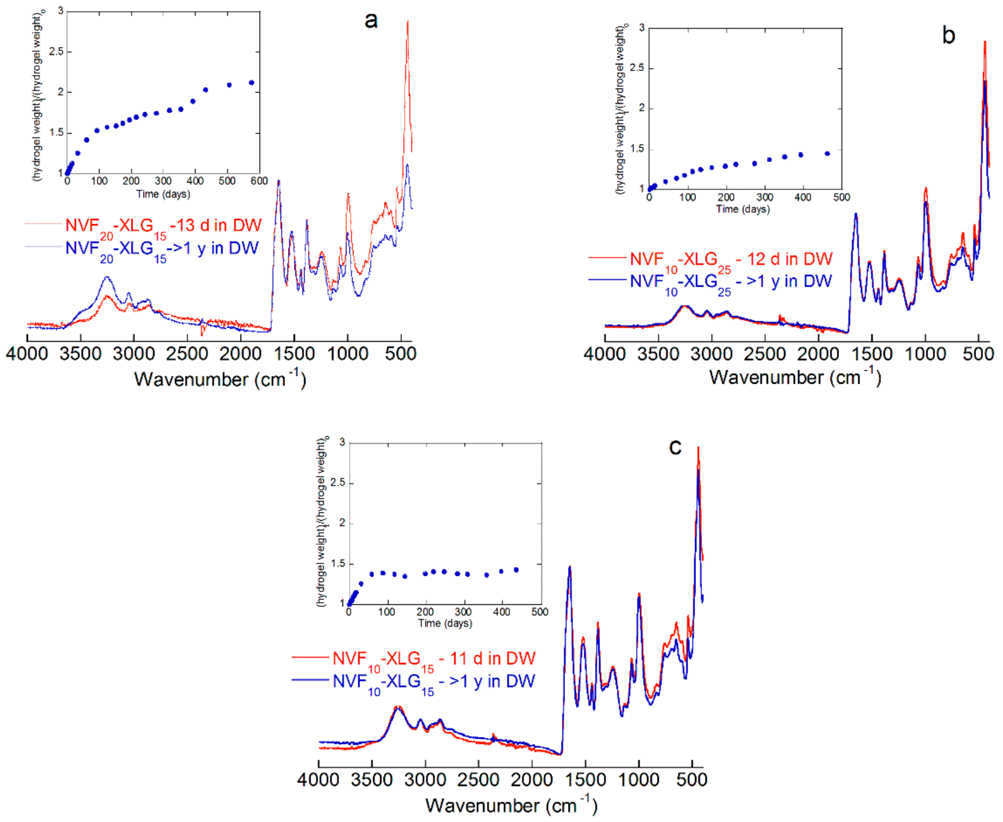

Figure S4. FTIR spectra of hydrogels stored in DW for more that one year in comparison with those of the corresponding hydrogels kept in DW for 11-13 days. a) NVF_{20}-XLG_{15}; b) NVF_{10}-XLG_{25}; c) NVF_{10}-XLG_{15}. All FTIR spectra were normalized against the band at ≈ 1650 cm^{-1}. The inset plots were built by dividing the hydrogel weight at time t to the initial weight of the hydrogel (after storing it in water for purification for several days after synthesis).

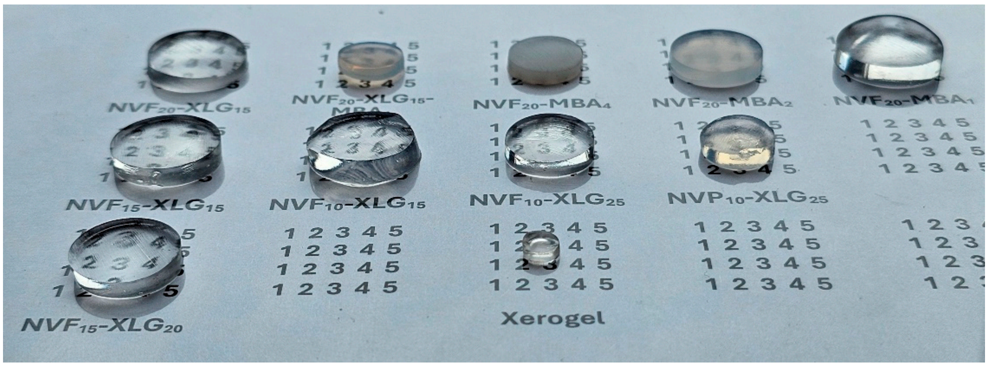

Figure S5. The thickness of the synthesized hydrogels after swelling in DW, at room temperature, for 7 days. Polished xerogels, with a thickness of 1±0.05 mm were employed in this experiment.

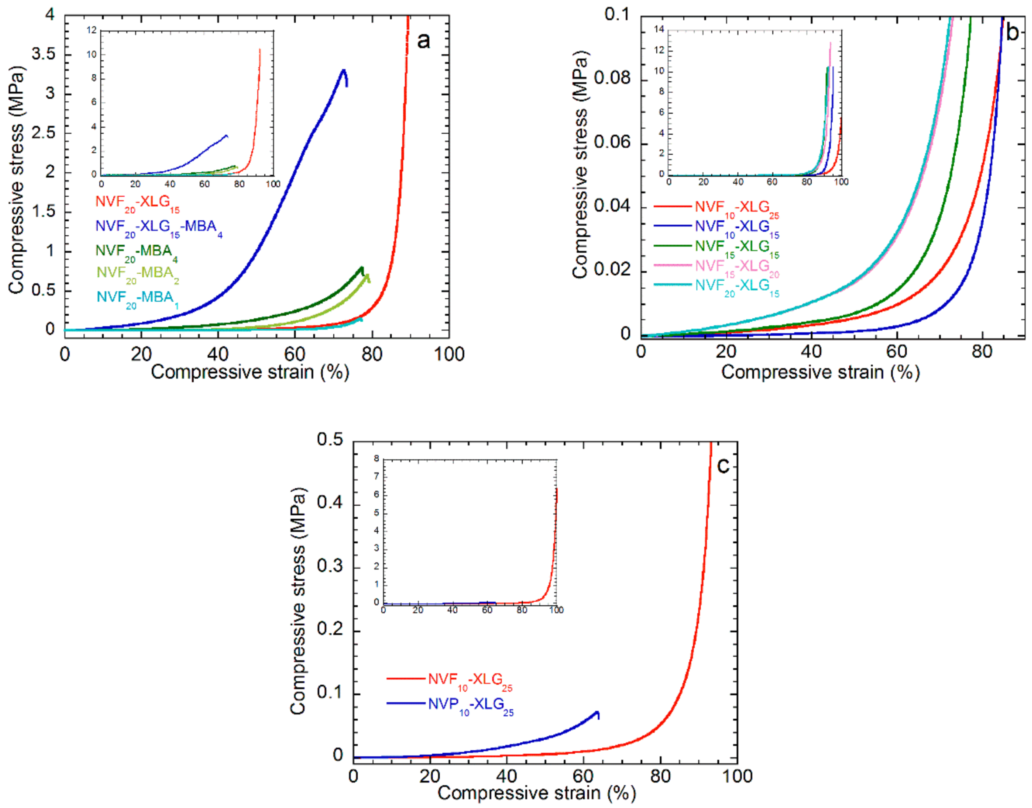

Figure S6. Typical compressive stress - strain curves for the hydrogels synthesized: a) comparison between the MBA-crosslinked hydrogels and NVF-XLG ones; b) typical stress-strain curves for the NVF-XLG nanocomposite hydrogels; c) influence of the monomer nature (NVP or NVF) on the shape of the stress-strain curves. The curves with the closest parameters to the average values shown in Table 1 are displayed.

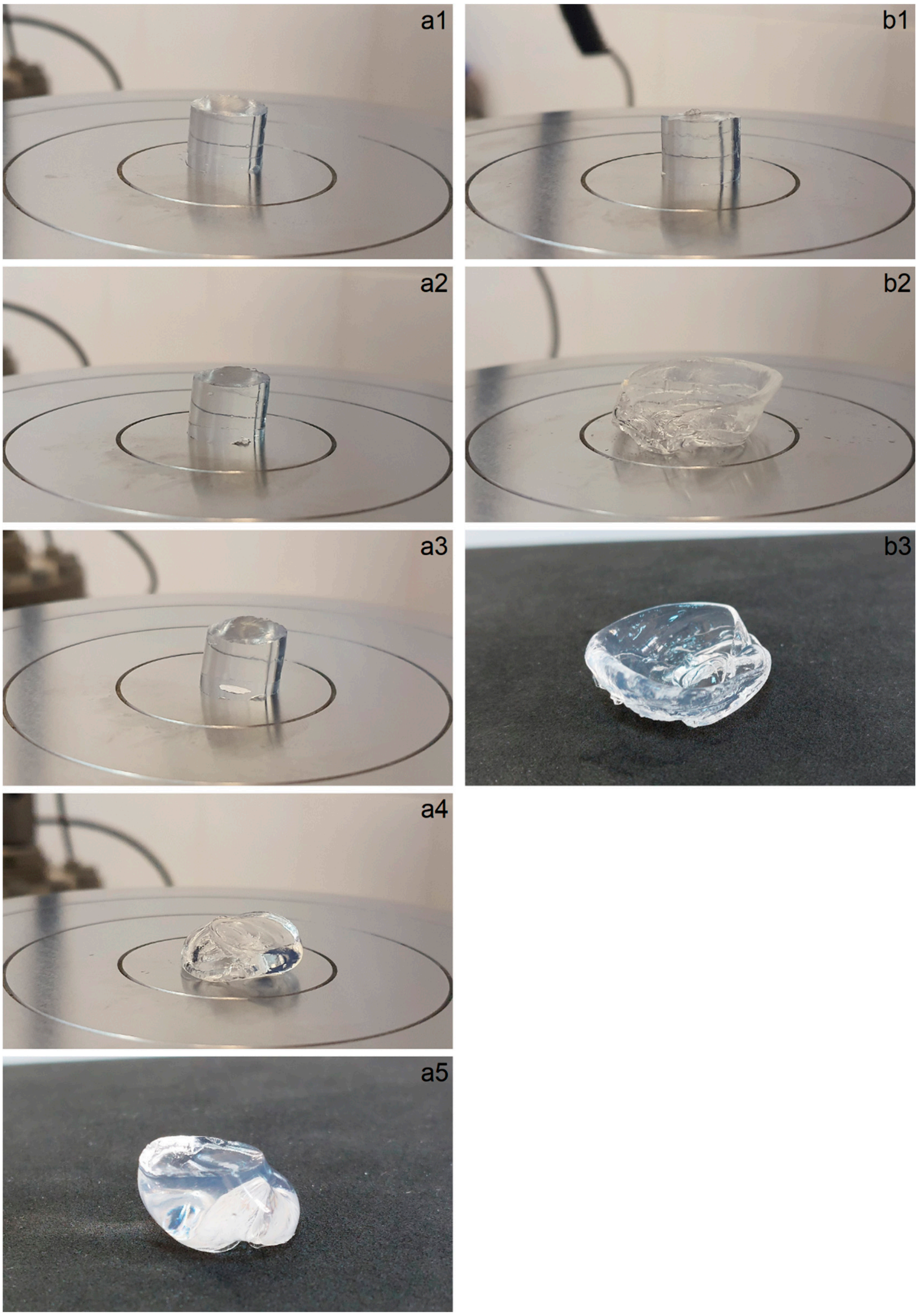

Figure S7. The shape of NVF_{15}-XLG_{20} nanocomposite hydrogel samples compressed at various strains. a1) initial; a2) after compression at 85% strain; a3) after compression at 90% strain; a4) after compression at 95% strain; a5) after re-swelling in DW at RT for 1 day. b1) initial; b2) compressed at 99% strain; b3) after re-swelling in DW at RT for 1 day.

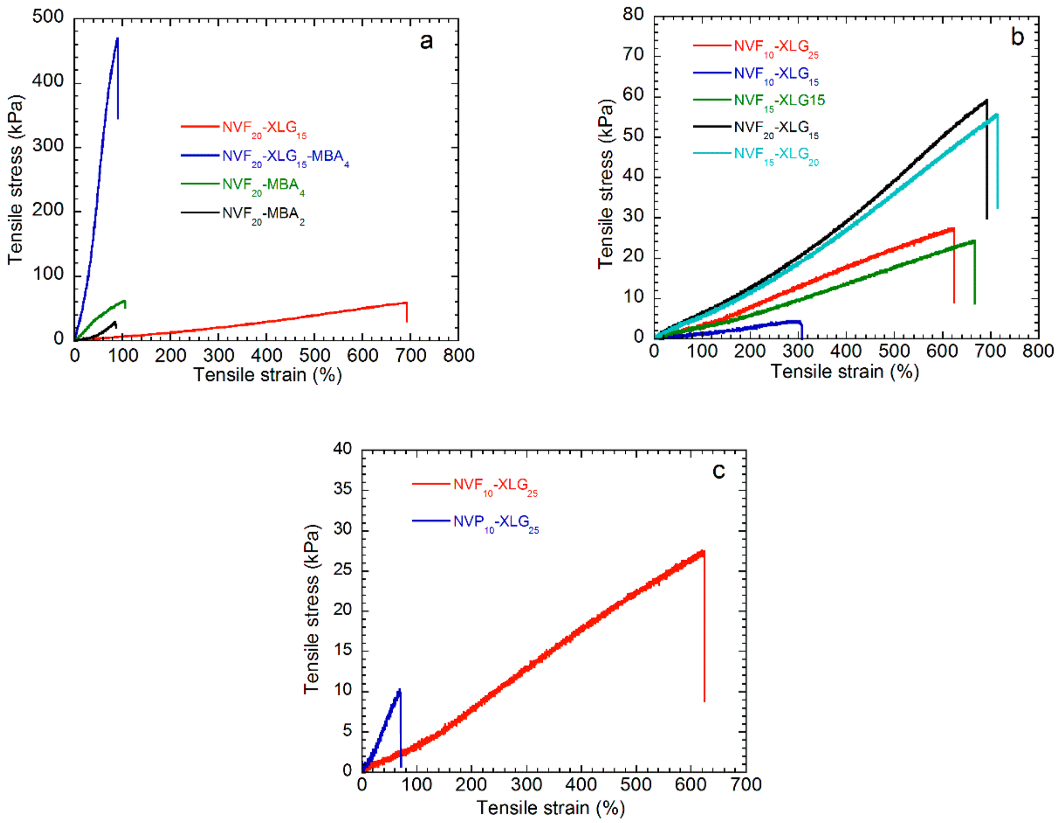

Figure S8. Typical tensile stress - strain curves for the hydrogels synthesized: a) comparison between the MBA-crosslinked hydrogels and NVF-XLG ones; b) typical stress-strain curves for the NVF-XLG nano-composite hydrogels; c) influence of the monomer nature (NVP or NVF) on the shape of the stress-strain curves. The curves with the closest parameters to the average values shown in Table 2 are displayed.

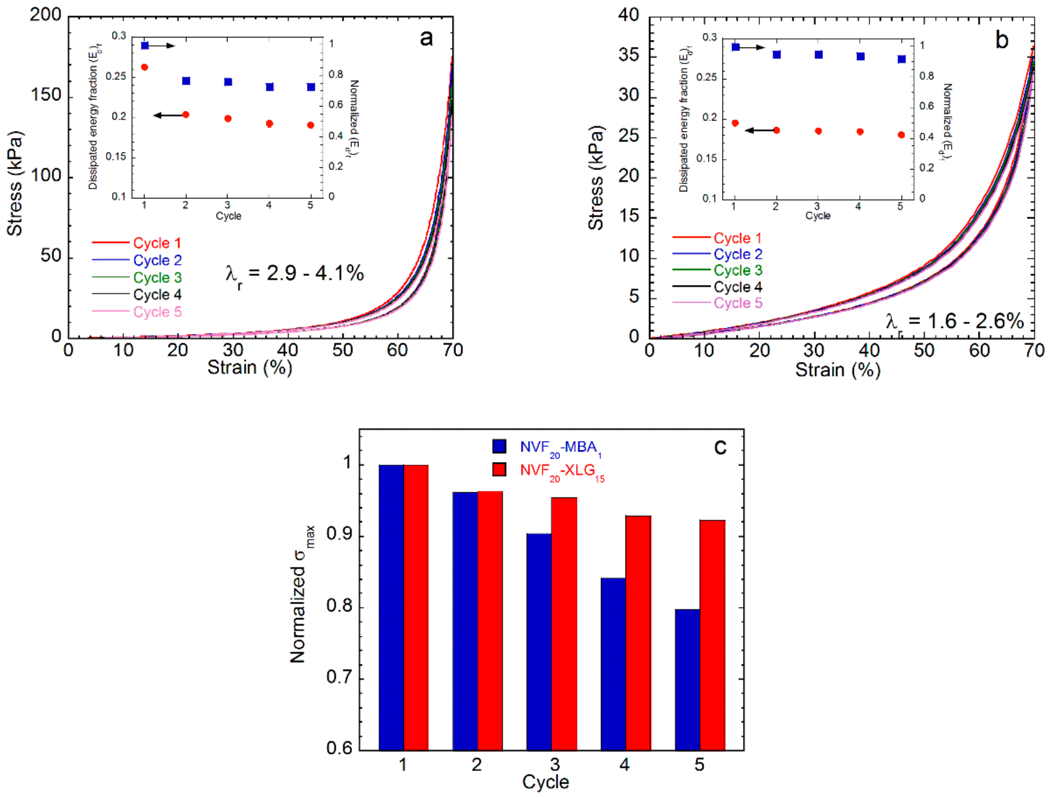

Figure S9. Cyclic compression tests: stress-strain curves for 70% maximum strain: comparison between the properties of NVF20-MBA1 and NVF20-XLG15. Stress-strain curves for 5 successive loading - unloading cycles, the corresponding residual strain λ_r range and the dissipated energy fraction $(E_d)_f$ (inset) in the case of a) NVF20-MBA1; b) NVF20-XLG15. c) The modification of the maximum stress (σ_{max}) as a function of the compression cycle.

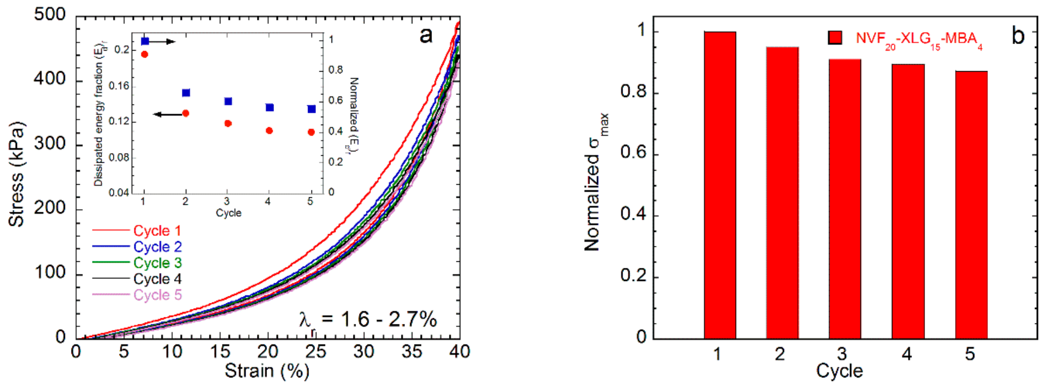

Figure S10. Cyclic compression tests in the case of NVF20-XLG15-MBA4. a) Stress-strain curves for 5 successive loading - unloading cycles, the corresponding residual strain λ_r range and the dissipated energy fraction $(E_d)_f$ (inset); b) The modification of the maximum stress (σ_{max}) as a function of compression cycle. Maximum strain = 40%. It matches the maximum stress that the instrument can apply.

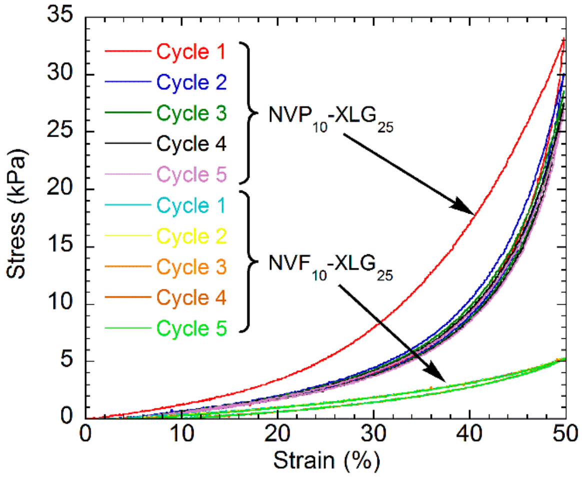

Figure S11. Cyclic compression tests: comparison between NVP10-XLG25 and NVF10-XLG25. Stress-strain curves for 5 successive loading - unloading cycles.

Table S1. Comparison of the elastic moduli obtained by compression (E_{comp}) and tensile (E_{tens}) tests, respectively.

Sample	SD^a g hydrogel/g xero-gel	E_{comp}^b kPa	E_{tens}^c kPa	SD^d g hydrogel/g xero-gel
NVP_{10}-XLG_{25}	22.3±0.5	13.9±0.2	12.3±4.0	21.8±0.1
NVF_{10}-XLG_{25}	34.0±1.2	4.3±0.1	5.0±2.5	35.1±0.9
NVF_{10}-XLG_{15}	57.9±3.6	1.2±0.1	2.3±0.8	58.9±1.0
NVF_{15}-XLG_{15}	33.1±0.2	5.6±0.1	4.8±1.6	37.8±0.9
NVF_{20}-XLG_{15}	24.1±0.2	13.8±0.3	9.0±2.5	28.7±0.5
NVF_{15}-XLG_{20}	24.6±1.1	13.4±1.0	10.3±1.0	26.7±0.3
NVF_{20}-XLG_{15}-MBA_4	7.6±0.3	338.2±9.5	288.5±9.2	8.0±0.1
NVF_{20}-MBA_4	15.1±0.1	70.9±1.1	62.4±5.1	13.9±0.1
NVF_{20}-MBA_2	28.2±0.1	14.4±0.3	13.0±2.0	27.6±0.1
NVF_{20}-MBA_1	40.0±0.1	5.7±0.1	-	-

[a]Swelling degree of the hydrogel at the time of compressive mechanical testing.

[b]Elastic modulus of the hydrogel determined by compressive mechanical tests.

[c]Elastic modulus of the hydrogel determined by tensile mechanical tests.

[d]Swelling degree of the hydrogel at the time of tensile mechanical testing.

Table S2. Confidence intervals (95% confidence level) calculated for the compressive and tensile mechanical properties of the hydrogels synthesized.

	**Compressive** mechanical properties - confidence interval -			**Tensile** mechanical properties - confidence interval -		
Sample	E_{comp}^a kPa	$\tau_{c,max}^c$ kPa	$(1-\lambda)_{max}^d$ %	E_{tens}^e kPa	$\tau_{t,max}^f$ kPa	$(\lambda-1)_{max}^g$ %
NVP_{10}-XLG_{25}	13.9±0.20 (±1.4%)	72.2±11.27 (±15.6%)	62.9±2.25 (±3.6%)	12.3±3.92 (±31.9%)	10.0±1.08 (±10.8%)	80.8±11.07 (±13.7%)
NVF_{10}-XLG_{25}	4.3±0.10 (±2.3%)	197.9±41.26 (±20.8%)	-	5.0±2.45 (±49.0%)	23.3±2.94 (±12.6%)	610.1±68.01 (±11.1%)
NVF_{10}-XLG_{15}	1.2±0.10 (±8.2%)	675.6±33.81 (±5.0%)	-	2.3±0.78 (±34.1%)	4.0±0.29 (±7.3%)	299.2±21.36 (±7.1%)

NVF_{15}-XLG_{15}	5.6±0.10 (±1.7%)	3765.7±1785.63 (±47.4%)	-	4.8±1.57 (±32.7%)	23.4±3.53 (±15.1%)	630.0±73.70 (±11.7%)
NVF_{20}-XLG_{15}	13.8±0.29 (±2.1%)	4552.1±1516.62 (±33.3%)	-	9.0±2.45 (±27.2%)	39.8±15.78 (±39.6%)	560.1±165.91 (±29.6%)
NVF_{15}-XLG_{20}	13.4±0.10 (±7.3%)	2189.4±764.88 (±34.9%)	-	10.3±0.98 (±9.5%)	56.4±8.82 (±15.6%)	843.7±203.54 (±24.1%)
NVF_{20}-XLG_{15}-MBA_4	338.2±9.31 (±2.8%)	3366.4±517.04 (±15.4%)	72.9±4.12 (±5.6%)	288.5±9.02 (±3.1%)	440.1±42.04 (±9.6%)	85.5±9.41 (±11.0%)
NVF_{20}-MBA_4	70.9±1.08 (±1.5%)	790.8±63.21 (±8.0%)	77.0±0.88 (±1.1%)	62.4±4.99 (±8.0%)	63.8±8.82 (±13.8%)	113.7±30.09 (±26.5%)
NVF_{20}-MBA_2	14.4±0.29 (±2.0%)	690.2±111.33 (±16.1%)	78.5±1.76 (±2.2%)	13.0±1.96 (±15.1%)	31.6±4.31 (±13.6%)	94.8±8.33 (±8.8%)
NVF_{20}-MBA_1	5.7±0.10 (±1.7%)	163.1±20.29 (±12.4%)	77.3±0.49 (±0.6%)	-	-	-

[a] Elastic modulus of the hydrogel determined by compression mechanical tests; [c] Ultimate compressive strength, in the case of MBA-containing hydrogels and NVP_{10}-XLG_{25} hydrogel, or the stress corresponding to 90% strain for the NVF-XLG nanocomposite hydrogels; [d] Ultimate compressive strain, in the case of MBA-containing hydrogels and NVP-XLG hydrogel, or 90%, in the case of NVF-XLG nanocomposite hydrogels; [e] Elastic modulus of the hydrogel determined by tensile mechanical tests; [f] Ultimate tensile strength; [g] Ultimate tensile strain.
